# Genome Sequence and Analysis of a Stress-Tolerant, Wild-Derived Strain of *Saccharomyces cerevisiae* Used in Biofuels Research

**DOI:** 10.1534/g3.116.029389

**Published:** 2016-04-16

**Authors:** Sean J. McIlwain, David Peris, Maria Sardi, Oleg V. Moskvin, Fujie Zhan, Kevin S. Myers, Nicholas M. Riley, Alyssa Buzzell, Lucas S. Parreiras, Irene M. Ong, Robert Landick, Joshua J. Coon, Audrey P. Gasch, Trey K. Sato, Chris Todd Hittinger

**Affiliations:** * Department of Energy (DOE) Great Lakes Bioenergy Research Center, University of Wisconsin-Madison, Wisconsin 53706; † Laboratory of Genetics, University of Wisconsin-Madison, Wisconsin 53706; ‡ Genome Center of Wisconsin, University of Wisconsin-Madison, Wisconsin 53706; § Wisconsin Energy Institute, J. F. Crow Institute for the Study of Evolution, University of Wisconsin-Madison, Wisconsin 53706; ** Microbiology Doctoral Training Program, University of Wisconsin-Madison, Wisconsin 53706; †† Department of Computer Sciences, University of Wisconsin-Madison, Wisconsin 53706; ‡‡ Department of Chemistry, University of Wisconsin-Madison, Wisconsin 53706; §§ Medical College of Wisconsin, Milwaukee, Wisconsin 53226; *** Department of Biochemistry, University of Wisconsin-Madison, Wisconsin 53706; ††† Department of Biomolecular Chemistry, University of Wisconsin-Madison, Wisconsin 53706

**Keywords:** lignocellulosic hydrolysates, Pacific Biosciences (PacBio), genome assembly, genome annotation, novel genes

## Abstract

The genome sequences of more than 100 strains of the yeast *Saccharomyces cerevisiae* have been published. Unfortunately, most of these genome assemblies contain dozens to hundreds of gaps at repetitive sequences, including transposable elements, tRNAs, and subtelomeric regions, which is where novel genes generally reside. Relatively few strains have been chosen for genome sequencing based on their biofuel production potential, leaving an additional knowledge gap. Here, we describe the nearly complete genome sequence of GLBRCY22-3 (Y22-3), a strain of *S. cerevisiae* derived from the stress-tolerant wild strain NRRL YB-210 and subsequently engineered for xylose metabolism. After benchmarking several genome assembly approaches, we developed a pipeline to integrate Pacific Biosciences (PacBio) and Illumina sequencing data and achieved one of the highest quality genome assemblies for any *S. cerevisiae* strain. Specifically, the contig N50 is 693 kbp, and the sequences of most chromosomes, the mitochondrial genome, and the 2-micron plasmid are complete. Our annotation predicts 92 genes that are not present in the reference genome of the laboratory strain S288c, over 70% of which were expressed. We predicted functions for 43 of these genes, 28 of which were previously uncharacterized and unnamed. Remarkably, many of these genes are predicted to be involved in stress tolerance and carbon metabolism and are shared with a Brazilian bioethanol production strain, even though the strains differ dramatically at most genetic loci. The Y22-3 genome sequence provides an exceptionally high-quality resource for basic and applied research in bioenergy and genetics.

Cellulosic bioethanol is a promising sustainable and renewable liquid transportation fuel (U.S. DOE 2006). Bioethanol is also a model fuel that is helping researchers understand the roadblocks involved in forcing cellular carbon flux away from biomass into toxic end-products, a challenge shared with advanced biofuels, including isobutanol and farnesene (Hong and Nielsen 2012; Buijs *et al.* 2013; U.S. DOE 2015). Although the yeast *Saccharomyces cerevisiae* has long been employed to convert starch sugars into ethanol, fermentation of sugars derived from the lignocellulose that makes up the cell wall of plants is more challenging. Due to its recalcitrant nature, lignocellulose-rich plant biomass, such as corn stover, must first be chemically, thermally, and/or mechanically pretreated to allow enzymes to efficiently hydrolyze cellulose and hemicellulose polymers into fermentable sugars. Although pretreatment methods can be effective at decreasing the hydrolysis time and increasing sugar yield, these methods often introduce toxic byproducts, including weak acids, amides, and aromatic compounds derived from the lignin itself; many of these compounds have potent negative effects on microbial fermentation (Piotrowski *et al.* 2014). In an attempt to mitigate the impacts of these and other stresses caused by fermentation, industrial *S. cerevisiae* strains have been selected for their robust tolerance phenotypes and further developed for lignocellulosic ethanol production, including the strains PE-2 (Pereira *et al.* 2014) and Ethanol Red (Demeke *et al.* 2013a). The genome sequences of several bioethanol production strains, including the PE-2-derivative JAY291 (Argueso *et al.* 2009), have been published, but the identities of the genes and variants that confer stress tolerance and other industrially desirable properties have generally remained unclear (Babrzadeh *et al.* 2012; Zheng *et al.* 2012; Sahara *et al.* 2014; Ulaganathan *et al.* 2015; Sravanthi Goud and Ulaganathan 2015).

In addition to the challenge of growth inhibitors from lignocellulosic hydrolysates, native *S*. *cerevisiae* is unable to ferment hemicellulosic pentose sugars, such as xylose, which constitute the second largest fraction of sugars in corn stover and most other plant biomass (Pauly and Keegstra 2008). Several groups have partially overcome these challenges by using strategies that combine rational engineering (*e.g.*, overexpressing genes encoding enzymes required for xylose fermentation) and directed evolution (*e.g.*, selecting for improved growth on xylose). These genetically modified strains of *S. cerevisiae* have a range of abilities to ferment the xylose present in lignocellulosic hydrolysates (van Maris *et al.* 2007; Koppram *et al.* 2012; Demeke *et al.* 2013a,b; Wei *et al.* 2013; Parreiras *et al.* 2014; Smith *et al.* 2014). Nonetheless, for evolved strains, it has often been unclear which mutations are responsible for the improved xylose fermentation.

The GLBRCY22-3 (Y22-3) yeast strain was developed to better understand the fermentation of xylose in lignocellulosic hydrolysates. Y22-3 is a monosporic derivative of NRRL YB-210 (YB-210), a wild strain of *S. cerevisiae* isolated from Costa Rican bananas (Mortimer and Johnston 1986). The YB-210 strain background was chosen for its unusual ability to tolerate high concentrations of ethanol (Wohlbach *et al.* 2014), elevated temperature, and the inhibitory compounds found in lignocellulosic hydrolysates made by two different types of alkaline pretreatment (Jin *et al.* 2013; Parreiras *et al.* 2014; Sato *et al.* 2014). In contrast, the standard S288c lab strain fares poorly under these stressful conditions. Although YB-210 does not utilize appreciable xylose natively, it was genetically engineered to express several heterologous enzymes required for efficient xylose metabolism; Y22-3 is one such haploid clone (Parreiras *et al.* 2014). Through the directed evolution of Y22-3 on xylose as the sole sugar source, a haploid clone, Y128, was isolated that could anaerobically ferment both glucose and xylose in Ammonia Fiber Expansion- (AFEX-) (Balan *et al.* 2009) pretreated corn stover hydrolysate (ACSH) (Parreiras *et al.* 2014).

Strains of *S. cerevisiae* and other species of *Saccharomyces* frequently contain genes not present in the S288c reference genome, especially in their subtelomeric regions (Liti and Louis 2005; Liti *et al.* 2009, 2013; Novo *et al.* 2009; Scannell *et al.* 2011; Borneman *et al.* 2011; Hittinger 2013; Bergström *et al.* 2014; Borneman and Pretorius 2015; Strope *et al.* 2015; Baker *et al.* 2015). These regions of yeast genomes are frequently laboratories of innovation where gene families expand, translocate, and evolve new functions (Carlson and Botstein 1983; Liti and Louis 2005; Hittinger 2013). Occasionally, genes are also added to these regions from other species by horizontal gene transfer (Novo *et al.* 2009; Dunn *et al.* 2012; Hittinger *et al.* 2015). Unfortunately, most whole genome shotgun sequencing strategies perform poorly on subtelomeric regions of the genome due to the widespread presence of selfish elements and polymorphic gene families with nearly identical sequences, leaving a blind (or at least blurry) spot in many genome assemblies where many of the most interesting and dynamic genes reside (Liti *et al.* 2009, 2013; Scannell *et al.* 2011; Borneman *et al.* 2011; Bergström *et al.* 2014; Strope *et al.* 2015; Baker *et al.* 2015). These genes can be responsible for novel traits (Borneman and Pretorius 2015), but investigation of these targets requires *de novo* genome sequencing strategies capable of obtaining refined genome assemblies with few gaps. Even for parts of the genome conserved with an essentially complete reference genome, such as S288c, the reliability of inferences from routine resequencing applications, such as RNA sequencing (RNA-Seq), copy-number variant (CNV) detection, and mutation inference, can be improved by mapping reads against a high-quality *de novo* assembly of the strain or line being studied (Pool 2015). Thus, a high-quality *de novo* assembly for Y22-3 is required to understand whether any novel genes have undergone mutations or changed their expression during its directed evolution into its more industrially relevant derivatives, such as the anaerobic xylose-fermenting strain Y128.

To enable functional genomic investigations of this emerging biofuel strain, we have assembled a high-quality reference genome for Y22-3. We benchmarked several genome assembly approaches, developed a genome assembly pipeline that integrated Pacific Bioscience (PacBio) sequencing reads with Illumina sequencing reads, and produced a fully annotated genome sequence. With few gaps in the nuclear genome, a complete mitochondrial genome, and a complete 2-micron plasmid sequence, the genome sequence of Y22-3 is among the highest quality *S. cerevisiae* genome sequences published. The Y22-3 genome has 92 nonrepetitive genes that S288c lacks, many of which are predicted to encode proteins whose functions are related to carbon metabolism or stress tolerance, including several that may be relevant to the strain’s tolerance to ACSH. Interestingly, although Y22-3 and the Brazilian bioethanol strain JAY291 are not closely related across most of their genomes, they share many genes that are rarely present in other strains. The Y22-3 genome sequence will provide an important foundation for basic and applied research.

## Materials and Methods

Complete details are available in Supplemental Material, File S1. Briefly, a single colony of Y22-3 genetically engineered for xylose metabolism (Parreiras *et al.* 2014) was grown in 10 g/L yeast extract, 20 g/L peptone and 20 g/L dextrose (YPD), and its genomic DNA was isolated and purified. The DNA sample was sequenced using the PacBio RS II technology with a C2 chemistry sequencing kit (Pacific Biosciences) to 155 × depth of coverage with an extracted subread length of 2881 ± 2177 bp and maximum read length of 35,845 bp (using *–minReadScore 0.75,–minLength 500* for pbh5tools, Pacific Biosciences, Menlo Park, CA), and by using the Illumina HiSeq technology with 100 bp paired-end reads with a raw depth of coverage of 1038 ×. An optimal assembly method was found by testing a variety of assembly methods that utilize either or both of the PacBio and Illumina data sets. Methods tested included the *de novo* assembly programs Sprai v. 0.9.9 (Imai 2013; Kamada *et al.* 2014), HGAP3 smart-analysis package v. 2.2.0.133377 (Chin *et al.* 2013), PBcR wgs-8.2beta (Koren *et al.* 2013), Velvet v. 1.2.10 (Zerbino and Birney 2008), and PBJelly (English *et al.* 2012), as well as the read preprocessing programs Trimmomatic (Bolger *et al.* 2014), Bless (Heo *et al.* 2014), and RACER (Ilie and Molnar 2013). Subsampling the paired-end reads down to 7% of the total number of trimmed reads was also examined. After testing the assembly methods, we assembled the nuclear genome and the 2-micron plasmid using Sprai v. 0.9.9 (Imai 2013; Kamada *et al.* 2014) and the mitochondrial genome using Spades v 3.5.0 (Nurk *et al.* 2013). We corrected single nucleotide polymorphisms (SNPs) and indels with Quiver (Chin *et al.* 2013) using the PacBio reads and with GATK v 3.1-1 (Van der Auwera *et al.* 2013) using the Illumina reads. We then annotated the nuclear and 2-micron assemblies by comparing, contrasting, and combining the predicted results from YGAP (Proux-Wéra *et al.* 2012) and Liftover (Kuhn *et al.* 2013). The mitochondrial assembly was first annotated using Liftover, followed by manual annotation using GENEIOUS v. R6 (Kearse *et al.* 2012).

We validated the predicted protein coding genes of Y22-3 using: 1) single-end RNA-Seq data collected from four growth phases of Y22-3 grown on YP media containing 60 g/L dextrose and 30 g/L xylose (YPDX, equivalent sugar concentrations that mimic ACSH made with 6% glucan loading), 2) an optimized (Figure S1) *de novo* transcriptome assembled by Trinity (Grabherr *et al.* 2011) using paired-end RNA-Seq data from clones derived from Y22-3 that were grown aerobically or anaerobically from four to six growth phases on YPDX and ACSH, and 3) proteomic data collected similarly to previous nanoflow liquid chromatography tandem mass spectrometry (nLC-MS/MS) approaches (Hebert *et al.* 2014) from Y22-3 cells grown aerobically in YPD. We compared the potentially novel genes of Y22-3 to other representative strains of *S. cerevisiae* using BLAST (Altschul *et al.* 1997) and developed a Novelty Metric to quantify how distinct non-S288c genes were from their nonsyntenic homologs in S288c. We examined the relationship between Y22-3 and other *S. cerevisiae* strains by generating a maximum likelihood phylogeny using RAxML v 8.1.20 (Stamatakis 2014) on an orthologous nucleotide dataset built from protein-coding sequences conserved across all strains.

### Data availability

This Whole Genome Shotgun project has been deposited at DDBJ/EMBL/GenBank under the accession LBMA00000000. The version described in this paper is version LBMA01000000. All DNA and RNA sequencing reads have been deposited in the NCBI SRA under BioProject PRJNA279877. Raw files for mass spectrometry data from these experiments are available on Chorus (https://chorusproject.org/pages/index.html, Project ID 999). Strains are available upon request. The authors state that all data necessary for confirming the conclusions presented in the article are represented fully within the article.

## Results and Discussion

### De novo genome assembly

**Figure 1:**
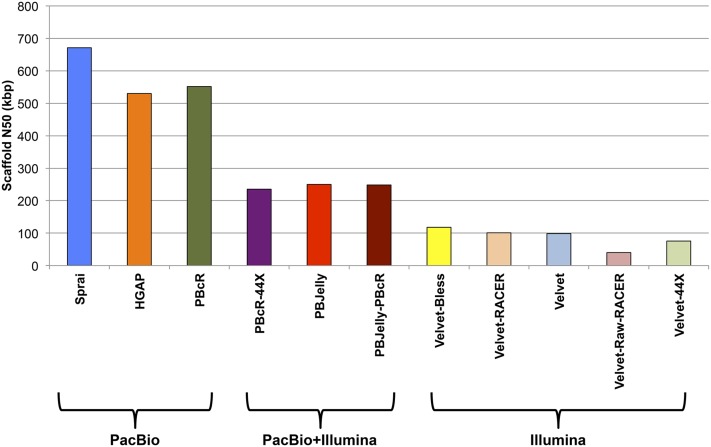
Scaffold N50 values obtained from various *de novo* assemblers with PacBio and paired-end Illumina reads. Note that, for the PacBio (Pacific Biosciences) assemblies, contig N50 values are equivalent to the scaffold N50 values.

To optimize assembly methods, we compared each strategy by their respective scaffold N50 values and found a wide range of performances (Figure 1). Strategies using only paired-end Illumina reads performed poorly. PBJelly, an algorithm that uses PacBio reads to scaffold Illumina-based assemblies, offered modest improvement in scaffolding. Error correction of PacBio using Illumina reads proved both computationally intensive and was outperformed by PBJelly on our dataset. Genome assemblies that were produced using exclusively self-corrected PacBio reads, including Sprai, HGAP, and PBcR, performed considerably better. Since Sprai achieved the best scaffold N50 and had the highest putative accuracy (Table S1), we continued to develop our pipeline with Sprai (Figure S2 and File S2).

We made several corrections to the Sprai assembly, including polishing with Quiver (Chin *et al.* 2013); three iterations of corrections using a custom GATK pipeline (Van der Auwera *et al.* 2013); ultra-scaffolding by homology to S288c (Figure S3); and special treatment of several regions, including to recover complete assemblies for the 2-micron plasmid and mitochondrial genome (Nurk *et al.* 2013; Baker *et al.* 2015). The N50 of the final ultra-scaffolded assembly of the nuclear genome was 908 kbp, and the contig (and scaffold) N50 was 693 kbp. Only nine gaps and 15 unplaced contigs remained, most of which contained fragments of Ty elements, whose full-length size of ∼6 kbp exceeded our average PacBio read length of 2.88 kbp. More than half of the chromosomes lacked any gaps, while chromosome XII contained the most gaps, including the one created by the *rDNA* repeats (Table S2).

### Genome annotation summary

To maximize the transfer of annotations from *S. cerevisiae* and related species of yeasts, we compared, contrasted, integrated, and improved on the results of two annotation pipelines: Liftover (Kuhn *et al.* 2013), which uses genome-wide alignment to a related genome, and the Yeast Genome Annotation Pipeline (YGAP), which features a *de novo* gene prediction algorithm and uses synteny and sequence similarity to infer homology (Proux-Wéra *et al.* 2012) (Figure S4). Using Liftover, we were able to transfer 6369 coding annotations from the S288c reference genome to the Y22-3 genome, of which 6004 were predicted to encode complete proteins. YGAP annotated 5820 genes, of which 5352 were predicted to encode complete proteins. We developed and applied an algorithm that corrected 123 of 365 Liftover annotations and 250 of 468 YGAP annotations, mainly by extending or shortening open reading frames (ORFs). After combining the annotations from Liftover and YGAP, manually correcting a few annotations, and manually correcting the mitochondrial annotations, we obtained 6319 valid coding gene annotations, 242 pseudogene annotations, and 297 tRNA annotations. The final annotated mitochondrial (Figure S5 and File S3) and nuclear genomes contained many features and genes not present in the S288c reference, including several that are rare or unique among strains with published genomes sequences.

### Validation of predicted genes using transcriptomic and proteomic data

To determine the impact that a nearly complete reference genome had on downstream functional genomic analyses, we compared the number of RNA-Seq reads mapped using the new Y22-3 reference genome, instead of the S288c reference genome. We observed a substantial increase in the fraction of RNA-Seq reads that could be mapped uniquely (83% *vs.* 78%), as well as a decrease in the number of reads that could not be mapped at all (Table 1). These results strongly suggest that the inclusion of novel genes and divergent alleles from Y22-3 is important for genomic applications based on read mapping.

**Table 1:** Impact of the reference genome used to map Y22-3 RNA-Seq reads

Reference Genome	Total Number of Reads	Trimmed Reads	Uniquely Mapped Reads (%)	Unmapped Reads (%)
S288c	139,272,379	114,570,068	89,851,385 (78%)	12,312,362 (11%)
Y22-3	139,272,379	114,570,068	94,708,638 (83%)	7,538,552 (7%)

**Figure 2:**
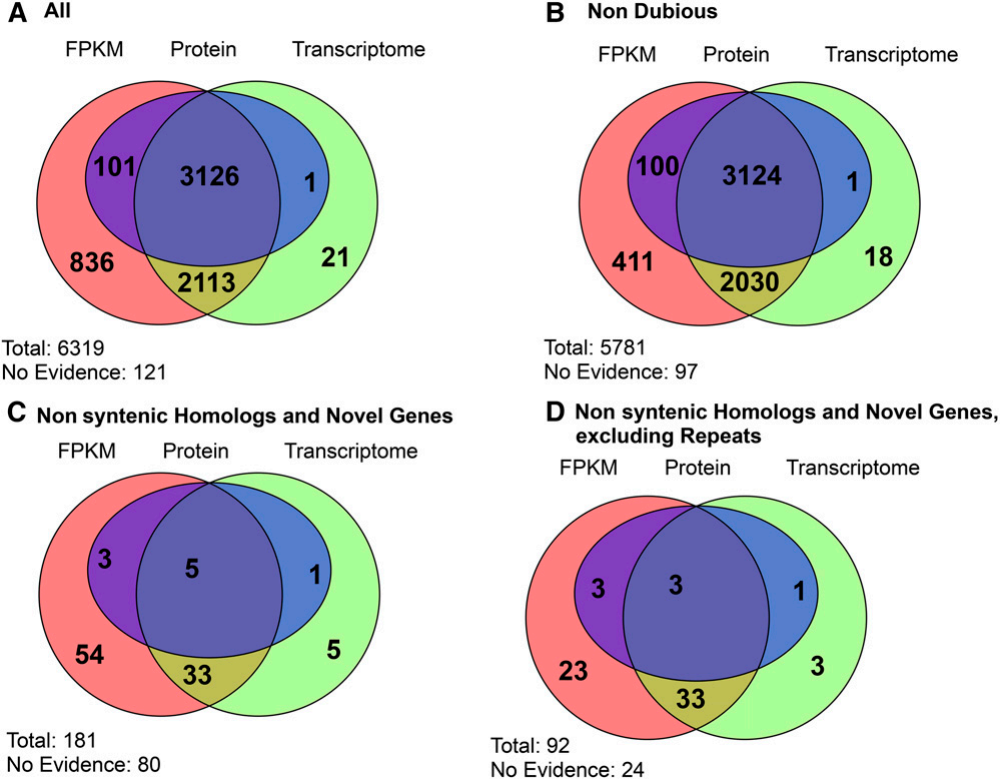
Venn diagram showing experimental evidence for annotated genes. Each number shows the overlap structure of the validations by transcriptome alignment (Transcriptome, green), transcript expression [“Fragments Per Kilobase of transcript per Million mapped reads” (FPKM), red], and proteins detected using mass spectrometry (Protein, blue). (A) Evidence for all protein-coding genes; (B) evidence for nondubious protein-coding genes; (C) evidence for protein-coding genes not present in S288c, including nonsyntentic homologs; and (D) evidence for genes not present in S288c, excluding transposons, helicases, and other subtelomeric repeats using RepeatMasker (Smit *et al.* 2013). Each figure also indicates the total number of genes (Total) and the number of genes for which no dataset validates their expression (No Evidence).

To validate the expression of predicted genes, we used transcriptomic and proteomic data to perform three different types of analyses (Figure 2, Table S3, and Table S4). First, we generated a *de novo* transcriptome assembly using 51 RNA-Seq experiments (Transcriptome Method). We considered a protein-coding gene validated if it had at least a 60% overlap with a predicted transcript that uniquely mapped to its locus. Second, we analyzed gene expression levels using eight RNA-Seq experiments (two replicates, four growth phases in YPD medium containing xylose, YPDX) (FPKM Method). We considered a protein-coding gene validated if it had an RNA expression value greater than 1 “Fragments Per Kilobase of transcript per Million mapped reads” (FPKM) in at least one experimental condition. Finally, a predicted protein was considered validated if one or more unique and unambiguously mapped peptides were detected by nLC-MS/MS (FDR < 0.01) (Protein Method).

Validations with individual methods ranged from 98.1% (6198/6319) for the FPKM Method to 51.1% (3228/6319) for the Protein Method (Figure 2). ORFs that were annotated as dubious by SGD (Cherry *et al.* 1998) were, perhaps not surprisingly, validated at considerably lower frequencies than ORFs that were not annotated as dubious [*e.g.*, for the Protein Method, 54.5% (3225/5920) *vs.* 0.5% (3/641), *P* < 10^−195^, Fisher’s Exact Test]. Even for genes not present in the S288c reference genome [excluding transposons, helicases, and other subtelomeric repeats detected using RepeatMasker (Smit *et al.* 2013)], we were able to validate 73.9% (68/92) by at least one method. Some of the genes not validated are the products of recent gene duplication events that cannot reliably be distinguished from one another.

### The Y22-3 genome lacks several genes relative to S288c

The annotated Y22-3 genome lacks 296 protein-encoding genes that are present in S288c (Table S5). Of the 296 missing genes, 139 are in subtelomeric regions in S288c (defined as within 50 kbp of the end of the assembled chromosome), and 156 are annotated as dubious ORFs. All five missing essential genes correspond to ORFs annotated as dubious, and prior experimental work in S288c suggests that deletion of these five dubious ORFs is lethal in S288c due to their effects on neighboring genes, rather than their intrinsic protein-coding potential (Engel *et al.* 2014). The assembly gap at the *rDNA* locus is responsible for 14 missing ORFs, while an assembly gap in the subtelomeric region of Chromosome II could explain the absence of four S288c ORFs, including two helicases, a dubious ORF, and a gene with no known function. Thus, we conclude that the missing genes are generally not assembly artifacts, but rather reflect differences in gene content. At least 22 of the missing genes have homologous genes on different chromosomes, suggesting that some of their functions may be performed by these nonsyntenic homologs. For example, Y22-3 appears to be missing *SOR1*, a gene encoding a sorbitol (and xylitol) dehydrogenase in S288c (Sarthy *et al.* 1994; Toivari *et al.* 2004; Wenger *et al.* 2010), but it retains the nearly identical paralog *SOR2*.

### The Y22-3 genome encodes several genes previously characterized in non-S288c strains

Several genes of interest for xylose metabolism (Wenger *et al.* 2010), stress tolerance, or other functions have been experimentally characterized in strains of *S. cerevisiae* other than S288c (Borneman and Pretorius 2015). Many of these genes have homologs in the Y22-3 genome, the S288c genome, or both. To quantify how distinct non-S288c genes are from their closest homolog in S288c, we developed a Novelty Metric to compare the strength of the best TBLASTN hit to the Y22-3 genome to the best TBLASTN hit to the S288c genome. Briefly, for each query gene, we subtracted the bit score generated against the S288c genome from the bit score generated against the Y22-3 genome (or any genome). We then normalized this value against the highest bit score generated against any *S. cerevisiae* genome in the dataset (see File S1, Equation 1). Thus, if a genome has a sequence that is closely related to a previously characterized non-S288c gene, it scores highly, while that genome scores poorly if it only has genes that are closely related to S288c homologs. Importantly, our Novelty Metric can recover homologs that are not annotated in the target genomes and quantifies how similar these sequences are to the non-S288c genes.

**Figure 3:**
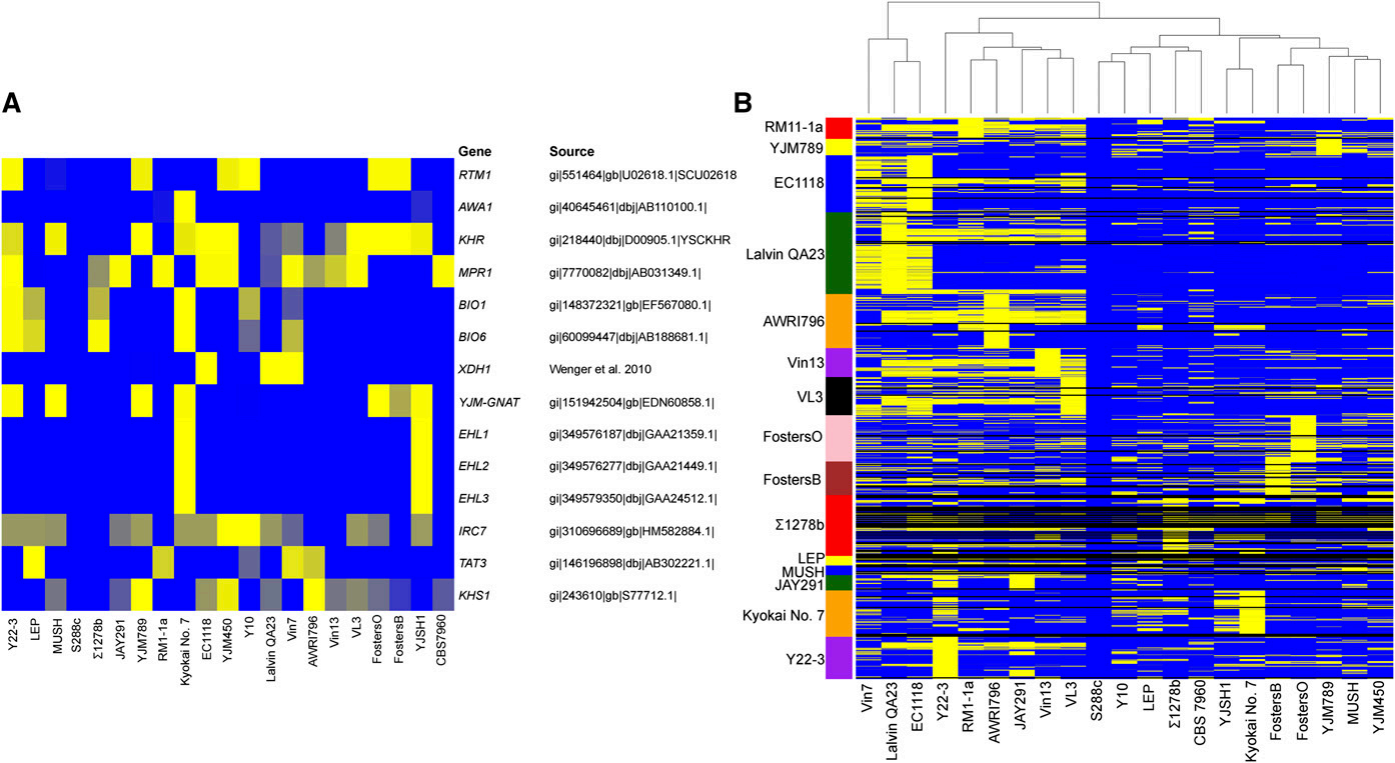
Heatmaps depicting the presence of previously characterized non-S288c genes and previously predicted ORFs not present in S288c. (A) Presence of previously characterized non-S288c genes; (B) previously predicted ORFs (open reading frames) not present in S288c; unsupervised clustering of the strains by gene content is shown above the heatmap. These heatmaps deploy our TBLASTN-derived Novelty Metric (File S1, Equation 1). Query genes are rows, and the genomes being searched are columns. A yellow value indicates a strong hit for a given query gene in that strain, whereas a blue value indicates a weak hit (or a hit similar to the best hit in the S288c genome). Note that, by definition, all values for S288c are zero (blue). Black values are not applicable. All strains listed are *S. cerevisiae*, except for Vin7, which is an allotriploid strain of *S. cerevisiae* × *S. kudriavzevii* (Borneman *et al.* 2012). Note that the *IRC7* gene used as the query gene was from strain YJM450 and may have been introgressed from *S. paradoxus* or another divergent lineage (Roncoroni *et al.* 2011).

Using our Novelty Metric, we found that Y22-3 contains several previously characterized genes that S288c lacks, many of which have roles in stress tolerance or metabolism that may be relevant to biofuel production (Figure 3A). These genes include *BIO1* and *BIO6*, two genes involved in biotin synthesis (Hall and Dietrich 2007); *RTM1*, which confers resistance to the toxicity of molasses, a substrate often used for industrial yeast biomass and ethanol production (Ness and Aigle 1995); *KHR1*, which encodes a heat-resistant killer toxin (Wei *et al.* 2007); *MPR1* or its close paralog, *MPR2*, which encodes a L-azetidine-2-carboxylic acid acetyl-transferase that can confer resistance to ethanol and freezing (Takagi *et al.* 2000); and *YJM-GNAT*, which encodes another N-acetyl-transferase (Wei *et al.* 2007). Critically, the Y22-3 genome does not encode *XDH1*, a gene encoding a xylitol dehydrogenase (Wenger *et al.* 2010) that could have interfered with the engineered xylose fermentation pathway. Although most have not been functionally characterized, many non-S288c ORFs have been predicted in other strains of *S. cerevisiae* (Argueso *et al.* 2009; Dowell *et al.* 2010; Borneman *et al.* 2011; Akao *et al.* 2011; Wohlbach *et al.* 2014), and our Novelty Metric suggests that Y22-3 also contains closely related homologs of some of these uncharacterized genes (Figure 3B).

### Several novel genes and gene clusters are predicted to encode functions related to stress tolerance and carbon metabolism

**Figure 4:**
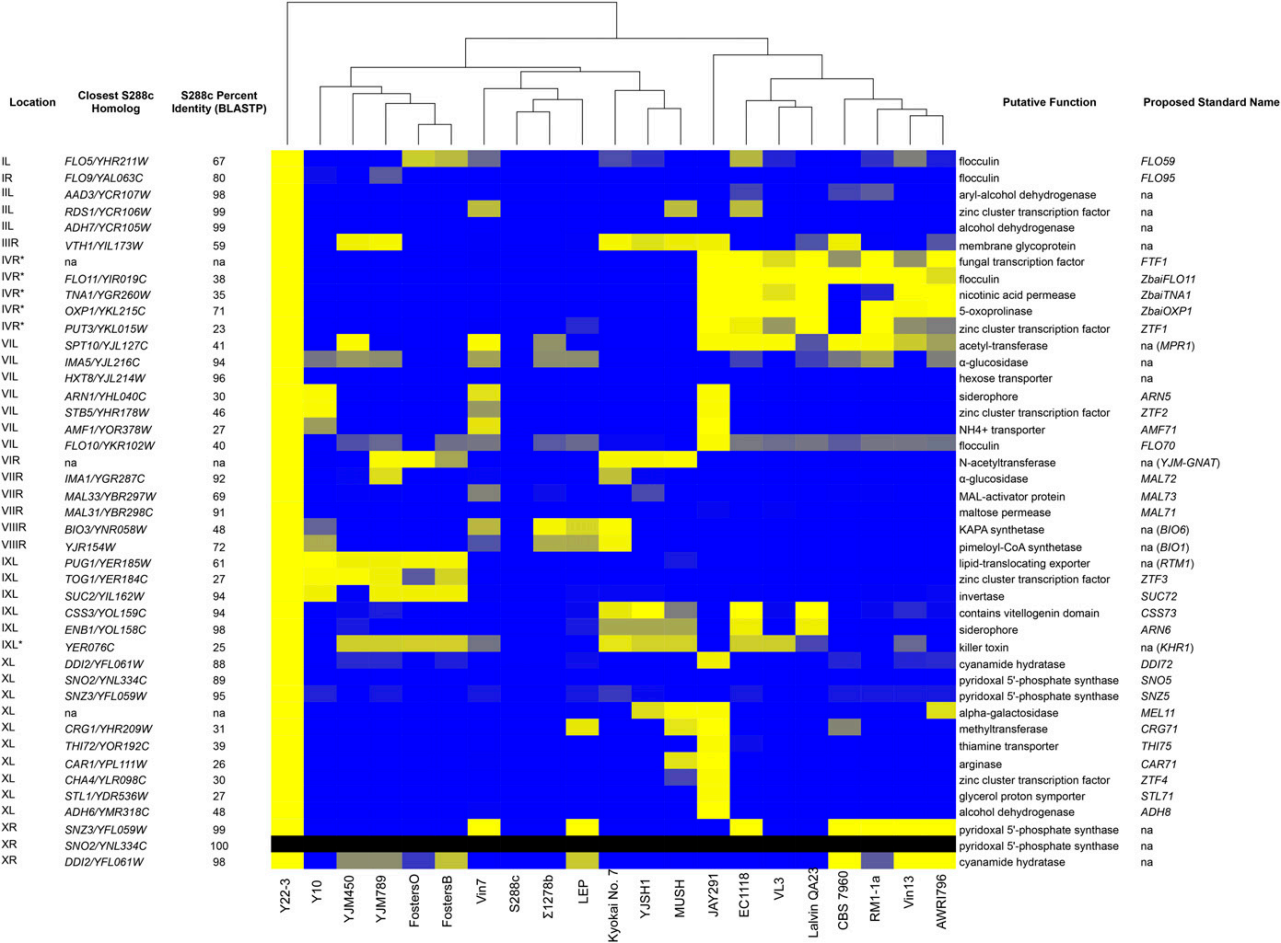
Novel genes and nonsyntenic homologs with functional annotations. The heatmap shows our TBLASTN-derived Novelty Metric (File S1, Equation 1) comparing the novel genes and nonsyntenic homologs found in Y22-3 against other strains of interest. A blue value indicates a strong hit for a given query gene in that strain, while a yellow value indicates a weak hit (or a hit similar to the best hit in the S288c genome). Black values are not applicable. Note that, by definition, all values for S288c are zero (blue). Unsupervised clustering of the strains by gene content is shown above the heatmap. Asterisks indicate nonsubtelomeric chromosomal locations; all other locations are subtelomeric. The closest S288c homolog is shown as not applicable (na) for genes where the best BLASTP hit had an e-value above 10^−3^. Standard names are proposed for 28 novel genes, while they are not proposed for 15 genes that match already named non-S288c genes or where they are the reciprocal best-BLAST hit of a S288c gene. Complete information for each gene, including the rationale for the proposed standard names, can be found in Table S6.

To further explore the genetic basis of the unusual stress tolerance and carbon metabolism properties of Y22-3, we closely examined 43 genes present in Y22-3 but not in S288c (Figure 4 and Table S6). For clarity, we did not consider repeat sequences that represent selfish elements (*e.g.*, Ty elements) and genes with no known functions [*e.g.*, *PAU* (Seripauperin) and *COS* genes] in the main manuscript (see Table S7 for full documentation). Expression of each of these 43 genes was detected in at least one condition by the FPKM Method (Table S6). Many are nonsyntenic homologs that are similar to well-characterized genes, whereas others are much more divergent, and their putative functional assignments are more tentative.

**Figure 5:**
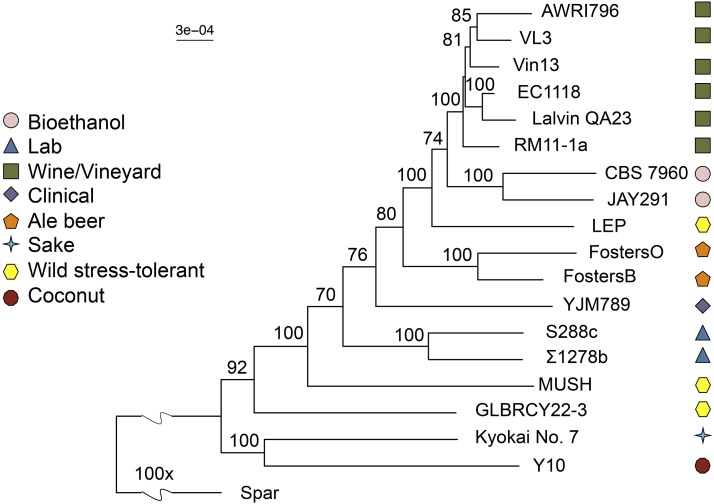
Genome-wide maximum likelihood phylogeny built using protein-coding nucleotide sequences. *S. paradoxus* was used as an outgroup. Bootstrap support values are to the left of their respective node. Note that the long terminal branch leading to GLBRCY22-3 is consistent with its previous assessment as a mosaic or admixed strain (Wohlbach *et al.* 2014). The scale is shown in substitutions per site, and the wavy line represents a 100 × scale discontinuity.

To quantify how novel these genes are, we again used our Novelty Metric to search for these genes in a panel of diverse strains with published genome sequences, as well as two other wild stress-tolerant strains (Birren *et al.* 2005; Wei *et al.* 2007; Argueso *et al.* 2009; Novo *et al.* 2009; Dowell *et al.* 2010; Borneman *et al.* 2011, 2012; Roncoroni *et al.* 2011; Akao *et al.* 2011; Zheng *et al.* 2012; Wohlbach *et al.* 2014; Fay *et al.* 2014a,b; Song *et al.* 2015). Most of these genes were found in a minority of the strains examined (Figure 4), suggesting that they could be at least partly responsible for some of the Y22-3 traits relevant to biofuel production. Interestingly, many of these genes are shared with another biofuel strain, JAY291 (Argueso *et al.* 2009), despite the fact that these strains are not phylogenetically closely related across most of their genome (Figure 5). This remarkable overlap advances the shared novel genes as particularly promising candidates for future studies investigating shared industrially relevant traits.

Several novel genes are predicted to encode functions related to stress tolerance, carbon metabolism, aldehyde or alcohol detoxification, and biofuel synthesis. A total of 28 genes with functional annotations were not syntenic and lacked reciprocal best-BLAST hits with S288c, and we have proposed standard names for them (Figure 4 and Table S6). For example, a homolog of *ADH6*, which encodes a cinnamyl alcohol dehydrogenase (Larroy *et al.* 2002), was especially divergent in sequence (48% maximum protein sequence identity), and we propose *ADH8* as its standard name. Since ferulic acid, *p*-coumaric acid, and related aromatic lignin degradation products are among the most toxic fermentation inhibitors in ACSH and many other lignocellulosic hydrolysates (Piotrowski *et al.* 2014), genes that reduce aromatic aldehydes into their less toxic alcohols may be beneficial. We also found two nonsyntenic homologs of *DDI2* and *DDI3*, which were recently shown to encode identical cyanamide hydratases in S288c (Li *et al.* 2015). If their activity is broader or the divergent (88% identical) homolog (*DDI72*) present in Y22-3 has novel activities, these genes might also metabolize other amides present in ACSH, such as acetamide, feruloyl amide, and *p*-coumaroyl amide (Chundawat *et al.* 2010).

The Y22-3 genome encodes several nonsyntenic homologs of genes involved in vitamin B1 (thiamine) and vitamin B6 metabolism. The novel gene *THI75* is distantly related (39% identical) to known thiamine transporters, while several additional genes are involved in the synthesis of pyridoxal 5′-phosphate, which is the active form of vitamin B6 (the novel genes *SNO5* and *SNZ5*, as well as additional nonsyntenic homologs of each). Previous studies on sugarcane bioethanol strains have found that increased copy numbers of *SNO* and *SNZ* genes improve growth in high sugar media lacking pyridoxine (vitamin B6) (Stambuk *et al.* 2009). Pyridoxal 5′-phosphate is a precursor for thiamine biosynthesis, and thiamine pyrophosphate is an obligate cofactor for many enzymes required for fermentation and the pentose phosphate pathway, including pyruvate decarboxylase and transketolase. The presence of additional copies of these genes in the Y22-3 genome suggests that similar constraints on vitamin B1 and B6 metabolism may also be important for lignocellulosic biofuel production.

**Figure 6:**
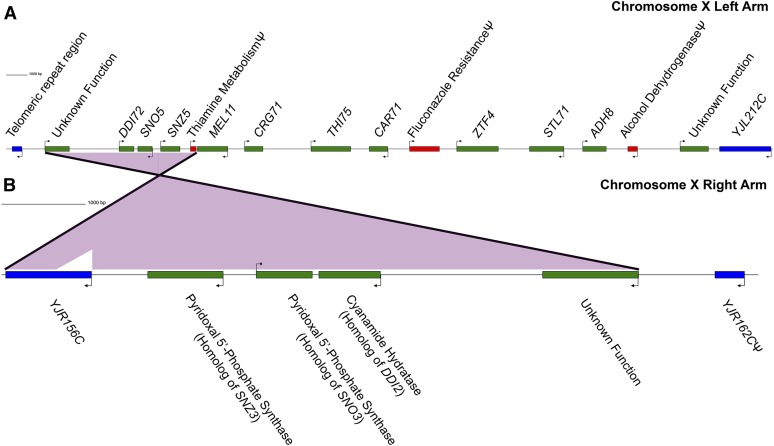
GenePalette (Rebeiz and Posakony 2004) depiction of chromosome X subtelomeric gene clusters. (A) Left-arm, (B) right-arm. Ψ, pseudogene. Features syntenic with S288c are in blue, novel genes and nonsyntenic homologs with valid coding regions are in green, and pseudogenes are in red. Synteny between the left and right arms is depicted by the purple triangles. The scale bars represent 1000 bp.

As is common in *S. cerevisiae* (Liti and Louis 2005; Liti *et al.* 2009; Strope *et al.* 2015), most (37/43) of these novel genes and nonsyntenic homologs mapped to subtelomeric regions, including an invertase (*SUC72*), an α-galactosidase (*MEL11*), several flocculins (*FLO59*, *FLO95*, and *FLO70*), and three Zn(II)_2_Cys_6_ transcription factors (*ZTF2*, *ZTF3*, and *ZTF4*). As is typically seen in *S. cerevisiae* (Liti and Louis 2005; Liti *et al.* 2009; Strope *et al.* 2015), most of the novel genes and nonsyntenic homologs are present in clusters (Figure 4 and Table S6). Ten clusters of two or more of these genes were found in Y22-3 (Figure 6, Figure S6, Figure S7, Figure S8, Figure S9, Figure S10, Figure S11, Figure S12, and Figure S13), but several clusters deserve special mention. One of the few nonsubtelomeric clusters is located in the interior of the right arm of chromosome IV and encodes a fungal transcription factor (*FTF1*), a flocculin (*ZbaiFLO11*), a nicotinic acid permease (*ZbaiTNA1*), an oxoprolinase (*ZbaiOXP1*), and a Zn(II)_2_Cys_6_ transcription factor (*ZTF1*) (Wohlbach *et al.* 2014; Parreiras *et al.* 2014) (Figure S7). These genes were also horizontally transferred from *Zygosaccharomyces bailii* into several wine strains (Novo *et al.* 2009) and apparently into Y22-3. The revised genome assembly presented here both completes and firmly places this and other clusters onto Y22-3 chromosomes, whereas the previous assembly often left such clusters incomplete and unplaced (Wohlbach *et al.* 2014). Many clusters include genes whose functions are likely related, such as the subtelomeric region of the right arm of chromosome VII, which includes a second complete maltose utilization cluster embedded within the *MAL1* cluster present in S288c; this novel cluster encodes a divergent isomaltase (*MAL72*), maltose transporter (*MAL71*), and activating Zn(II)_2_Cys_6_ transcription factor (*MAL73*) (Figure S10). The interior of the right arm of chromosome VIII contains at least six copies of *CUP1* with a spacing consistent with the recently described Type 3 (Zhao *et al.* 2014) configuration (Figure S11); the locus could also contain additional copies because no PacBio reads fully spanned the repeats. Most strikingly, both the left and right subtelomeric regions of chromosome X contain clusters of genes related to thiamine metabolism and encoding amide hydratases (Figure 6). The nonsyntenic homologs of the genes present in the right subtelomeric region of chromosome X are relatively closely related to genes present on the left subtelomeric region of chromosome VI in S288c, while those in the left subtelomeric region of chromosome X appear to be highly divergent in Y22-3 and are often shared only with the bioethanol strain JAY291 (Figure 4).

### Conclusions

Here, we have developed a genome assembly pipeline that integrates PacBio and deep Illumina paired-end sequencing coverage. The Y22-3 genome sequence assembled is one of the highest quality *S. cerevisiae* genome sequences published. Most nuclear chromosomes are complete, including several challenging regions, such as subtelomeric regions. The mitochondrial genome and 2-micron plasmid sequences are complete. Careful annotation revealed several novel genes and gene clusters, many of which have predicted roles in stress tolerance or fermentation. Genes involved in thiamine metabolism, involved in carbon metabolism, encoding enzymes that act on aromatic lignin degradation products, and encoding amidases, are likely to be particularly relevant for biofuel production by Y22-3 in ACSH and other lignocellulosic hydrolysates. Strikingly, many closely related genes are also found in the genome of the Brazilian bioethanol strain JAY291, suggesting that there may be a common genetic basis for some of their industrially relevant properties. The complete genome sequence of Y22-3 will enable ongoing and future investigations into its novel properties, including approaches using molecular genetics, functional genomics, and directed evolution.

## 

## Supplementary Material

Supplemental Material
