# Supplementary material for: Genome Sequence and Analysis of a Stress-Tolerant, Wild-Derived Strain of Saccharomyces cerevisiae Used in Biofuels Research
Source: G3 (Bethesda). 2016 Apr 16;6(6):1757–66. doi: 10.1534/g3.116.029389 (PMC4889671; doi:10.1534/g3.116.029389)
Supplement: Supplemental Material [file supp_6_6_1757__index.html]

Genome Sequence and Analysis of a Stress-Tolerant, Wild-Derived Strain of Saccharomyces cerevisiae Used in Biofuels Research — Supplemental Material 

# Genome Sequence and Analysis of a Stress-Tolerant, Wild-Derived Strain of *Saccharomyces cerevisiae* Used in Biofuels Research

## Supplemental Material for McIlwain *et al.*, 2016

**Files in this Data Supplement:**

- Figure S1 - A sample region from chromosome XIV demonstrating the predicted genes (blue track), the alignment of the predicted transcripts generated with default-parameter Trinity assembly (grey zone), and the alignment of the predicted transcripts generated with the optimized Trinity assembly (green zone). (.pdf, 105 KB)
- Figure S10 - GenePalette depiction of novel genes and non-syntenic homologs found in the subtelomeric region of the right arm of chromosome VII. (.pdf, 51 KB)
- Figure S11 - GenePalette depiction of the *CUP1* locus found on chromosome VIII. (.pdf, 34 KB)
- Figure S12 - GenePalette depiction of novel genes and non-syntenic homologs found in the subtelomeric region of the right arm of chromosome VIII. (.pdf, 32 KB)
- Figure S13 - GenePalette depiction of novel genes and non-syntenic homologs found in the subtelomeric region of the left arm of chromosome IX. (.pdf, 57 KB)
- File S1 - Supplementary materials and methods. (.pdf, 206 KB)
- File S2 - Genome assembly scripts. (.pdf, 40 KB)
- File S3 - Supplementary results. (.pdf, 57 KB)
- Figure S2 - Flowchart of the steps taken to generate the final de novo assembly of Y22-3. (.pdf, 50 KB)
- Figure S3 - Dot plots for each chromosome using S228c as the reference on the x-axis and the Y22-3 assembly on the y-axis. (.pdf, 623 KB)
- Figure S4 - Y22-3 genome annotation pipeline. (.pdf, 12 KB)
- Figure S5 - Schematic representation of A) Y22-3 and B) S288c mitochondrial genome annotations. (.pdf, 225 KB)
- Figure S6 - GenePalette depiction of novel genes and non-syntenic homologs found in the subtelomeric region of the left arm of chromosome II. (.pdf, 75 KB)
- Figure S7 - GenePalette depiction of novel genes and non-syntenic homologs found on chromosome IV. (.pdf, 39 KB)
- Figure S8 - GenePalette depiction of novel genes and non-syntenic homologs found in the subtelomeric region of the left arm of chromosome VI. (.pdf, 56 KB)
- Figure S9 - GenePalette depiction of novel genes and non-syntenic homologs found in the subtelomeric region of the right arm of chromosome VI. (.pdf, 38 KB)
- Table S1 - Assessment of the qualities of PacBio assemblies with QUAST. (.xlsx, 11 KB)
- Table S2 - Genome assembly summary. (.xlsx, 9 KB)
- Table S3 - Gene validation by expression. (.xlsx, 333 KB)
- Table S4 - Unique peptides detected by nLC-MS/MS. (.xlsx, 2181 KB)
- Table S5 - S288c genes missing the final genome assembly of Y22-3. (.xlsx, 21 KB)
- Table S6 - Detailed information on novel genes and non-syntenic homologs with functional annotations, including proposed standard names. (.xlsx, 17 KB)
- Table S7 - Systematic BLAST results for all novel genes and non-syntenic homologs. (.xlsx, 305 KB)
