# Supplementary material for: Genome Sequence and Analysis of a Stress-Tolerant, Wild-Derived Strain of Saccharomyces cerevisiae Used in Biofuels Research
Source: G3 (Bethesda). 2016 Apr 16;6(6):1757–66. doi: 10.1534/g3.116.029389 (PMC4889671; doi:10.1534/g3.116.029389)
Supplement: Supplemental Material [file supp_g3.116.029389_FigureS11.pdf]

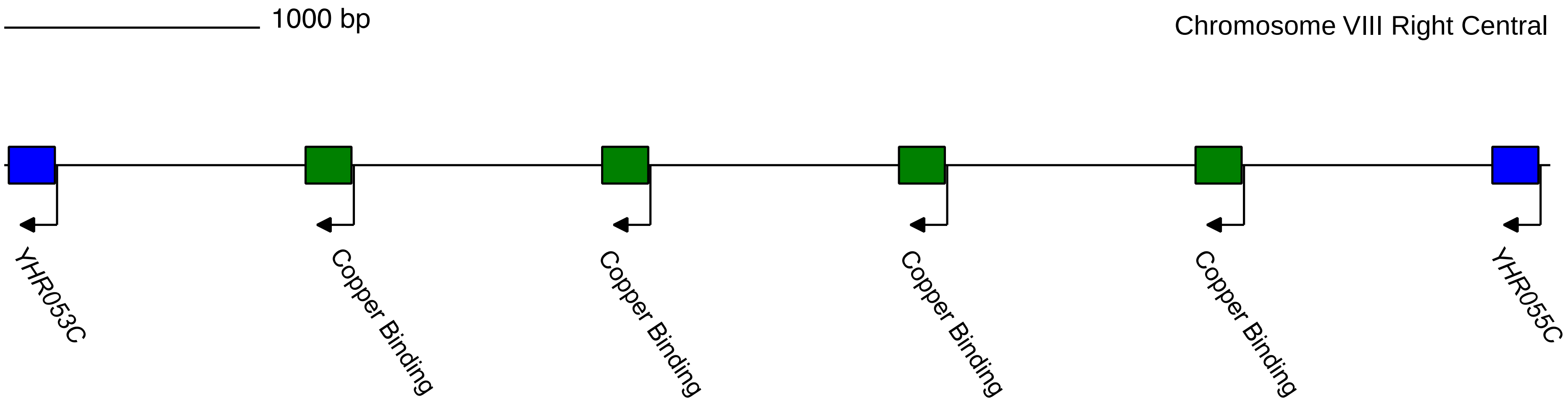

Figure S11. GenePalette depiction of the *CUP1* locus found on chromosome VIII. Features syntenic with the S288c reference genome are in blue, while extra copies of *CUP1* are depicted in green. The scale bar represents 1000 bp.
