# Supplementary material for: Genome Sequence and Analysis of a Stress-Tolerant, Wild-Derived Strain of Saccharomyces cerevisiae Used in Biofuels Research
Source: G3 (Bethesda). 2016 Apr 16;6(6):1757–66. doi: 10.1534/g3.116.029389 (PMC4889671; doi:10.1534/g3.116.029389)
Supplement: Supplemental Material [file supp_g3.116.029389_FileS1.pdf]

FILE S1: SUPPLEMENTARY MATERIALS AND METHODS

Isolation of Y22-3 genomic DNA: The genetic background and genetic engineering of GLBRCY22-3 (Y22-3) have been described elsewhere (Parreiras *et al.* 2014). Briefly, Y22-3 is a haploid *MAT***a** derivative of YB-210, which has been engineered for xylose utilization. A single colony of Y22-3 strain was cultured in 500 mL 10 g/L yeast extract, 20 g/L peptone, and 20 g/L dextrose (YPD) media at 30 ^{o}C overnight. Cells were centrifuged at 3,000 relative centrifugal force (RCF) for 15 min at 4 ^{o}C. The resulting cell pellet was washed with 10 mM Tris, 0.5 mM EDTA, pH 8.0 and centrifuged. The washed cell pellet was then resuspended in 30 mL of 50 mM EDTA and incubated with 1,000 U Zymolyase at 30 ^{o}C for 1 hr. The resulting spheroplasts were centrifuged at 3,000 RCF at 4 ^{o}C for 15 min; completely resuspended in 15 mL sterile 0.2 M Tris, pH 8.5, 0.25 M NaCl, 25 mM EDTA, 0.5% sodium dodecylsulfate, 3 mg RNase A, 8 mg Proteinase K; and incubated at 50 ^{o}C for 30 min. Cell lysate was then clarified by centrifugation; extracted with 10 mL Tris-equilibrated phenol, pH 8.0; and incubated at room temperature for 20 min. The phenol-lysate mixture was then centrifuged at 3,000 RCF for 15 min, and the upper aqueous phase was transferred to a sterile 50 mL conical tube. The aqueous phase was subsequently extracted twice with 10 mL chloroform, as done with the phenol. To the final phenol/chloroform-extracted aqueous phase, 1/10th of the total volume of 3 M sodium acetate and two volumes of isopropanol were added and mixed by inversion. DNA was allowed to precipitate by incubation at -80 ^{o}C for 20 min and pelleted by centrifugation at 5,000 RCF for 30 min at 4 ^{o}C. The DNA pellet was washed with ice-cold 70% ethanol, air dried for 15 min, and then dissolved in 10 mM Tris, 0.5 mM EDTA, pH 8.0 (TE buffer). Purified genomic DNA

(gDNA) was then further column-purified (Genomic Tip-500, Qiagen) to a final concentration of 60 ng/μL in 200 μL TE buffer.

Pacific Biosciences library preparation and sequencing: The isolated gDNA was submitted to the Medical College of Wisconsin for PacBio sequencing. Initial quantification was performed using a Quant-iT™ PicoGreen® dsDNA Assay Kit (Life Technologies, Carlsbad, CA, USA) and a NanoDrop 2000 (Thermo Fisher Scientific Inc., Waltham, MA, USA). gDNA was purified and concentrated with a 0.45x AMPure PB bead wash (Pacific Biosciences, Menlo Park, CA, USA). About 5 μg of concentrated gDNA was sheared to 10 kbp using a Covaris gTube (Covaris Inc, Woburn, MA, USA). A Pacific Biosciences (PacBio) sequencing library, or SMRTbell™, was constructed using the SMRTbell™ Template Prep Kit 1.0 and the 10 kbp Template Preparation and Sequencing with Low-Input DNA procedure (Pacific Biosciences). P4 Polymerase was coupled with the resulting SMRTbell™ library. For sequencing, the library was bound to MagBeads by incubating for 1 hr at 4 °C. The final library was run over 8 V2 SMRT cells on the PacBio RSII using a C2 chemistry sequencing kit (Pacific Biosciences). Each SMRT cell was visualized using a 1x180 min movie. The PacBio subreads were extracted using pbh5tools (PacificBiosciences, Menlo Park, CA, USA) with --*minReadScore 0.75* and *--minLength 500*.

Illumina library preparation and sequencing: The isolated gDNA was submitted to the University of Wisconsin Biotechnology Center (UWBC) DNA Sequencing Facility for Illumina sequencing. DNA concentration and sizing were verified using the Qubit® dsDNA HS Assay Kit (Life Technologies, Grand Island, NY) and Agilent DNAHS chip (Agilent Technologies, Inc., Santa Clara, CA), respectively.

Samples were prepared according the TruSeq® Genomic DNA Sample Preparation kit v2 (Illumina Inc., San Diego, California, USA) with minor modifications. Samples were sheared using either a Diagenode Bioruptor (Diagenode USA, Denville, NJ) or a Covaris M220 Ultrasonicator (Covaris Inc, Woburn, MA, USA). Libraries were size-selected for an average insert size of 275 bp using a 2% Invitrogen E-Gel (Life Technologies). Quality and quantity of the finished libraries were assessed using an Agilent DNA1000 chip and Qubit® dsDNA HS Assay Kit, respectively. Cluster generation was performed using standard Cluster Kits and the Illumina Cluster Station. Paired-end, 100-bp sequencing was performed using standard SBS chemistry on an Illumina HiSeq 2000 sequencer. Images were analyzed using the standard Illumina Pipeline, version 1.8.2.

Optimization of the de novo genome assembly: Many de novo genome assembly pipelines can utilize long but error-prone PacBio reads and/or short Illumina reads. Some pipelines can leverage the two, either by using the Illumina data to correct the sequencing errors in the PacBio data or by scaffolding prior de novo assemblies using the PacBio data. Methods also exist to self-correct Illumina data prior to assembly (Ilie and Molnar 2013; Heo *et al.* 2014). To determine the optimal genome assembly method, we tested several de novo genome assembly and read preprocessing algorithms.

Using only PacBio reads, we ran Sprai v. 0.9.9 (Imai 2013; Kamada *et al.* 2014), HGAP3 smart-analysis package v. 2.2.0.133377 (Chin *et al.* 2013), and PBcR wgs-8.2beta (Koren *et al.* 2013) using their default or provided parameters. Using only

paired-end Illumina reads, we created assemblies using Velvet v. 1.2.10 (Zerbino and Birney 2008) with a series of different *kmer* sizes {45, 47, … 99}. In addition to using raw reads, we also tried preprocessing the reads using Trimmomatic (Bolger *et al.* 2014), Bless (Heo *et al.* 2014), and RACER (Ilie and Molnar 2013). We also tried subsampling the paired-end reads down to 7% of the total number of trimmed reads (6,121,566/87,466,528 or about 44X/625X trimmed coverage). Finally, we ran RACER with and without prior trimming with Trimmomatic (Bolger *et al.* 2014). PBcR (Koren *et al.* 2013) allows for the use of Illumina reads to correct the PacBio reads prior to assembly using Celera (Myers *et al.* 2000). Unfortunately, computational limitations prevented us from using all of the Illumina data, so we again reduced the number of reads to 7% (PBcR-44X). We also used PBJelly (English *et al.* 2012) to scaffold PBcR (PBJelly-PBcR) and Velvet (PBJelly) assemblies using the PacBio reads. The accuracy of the Sprai, HGAP, and PBcR assemblies was assessed using QUAST v3.2 build 26.11.2015 (Gurevich *et al.* 2013).

Although the contig (and scaffold) N50 of the Sprai assembly was of exceptionally high continuity at 671 kbp, polishing our assembly with Quiver (Chin *et al.* 2013) detected and corrected 1359 SNPs and 1073 indels (247 deletions, and 826 insertions). We then removed redundant contigs with the check_redundancy.pl script included in the Sprai package and ordered the remaining contigs by aligning homologous regions to the S288c genome. After the putative order was determined, adjacent contigs were checked for possible overlaps using Staden (Bonfield and Whitwham 2010), a process that replaced eight gaps with a consensus sequence. Contigs that could not be joined were then ultra-scaffolded by homology to S288c using

a gap with an arbitrary size of 5 kbp. A handful of small inversions were detected using dot plots built with Gepard (Krumsiek *et al.* 2007), but no major non-subtelomeric translocations were detected relative to S288c (**Figure S3**); we cannot definitively exclude the possibility that translocations exist at the few gaps remaining in our assembly. We trimmed and reoriented the complete 2-micron plasmid to match the orientation of S288c. Sprai performed poorly on the mitochondrial genome, so we assembled the Sprai-corrected PacBio reads and paired-end Illumina reads into the complete mitochondrial genome with Spades v 3.5.0 (Bankevich *et al.* 2012), which was recently shown to yield complete *Saccharomyces* mitochondrial assemblies from paired-end Illumina reads alone (Baker *et al.* 2015). We iteratively deployed GATK v 3.1-1 (Van der Auwera *et al.* 2013) to correct an additional 9 SNPs and 217 indels with the Illumina paired-end data, reaching convergence after three iterations.

After selecting Sprai as the optimal de novo assembler, additional single nucleotide polymorphisms (SNPs) and indels were found with GATK v 3.1-1 (Van der Auwera *et al.* 2013) using the Illumina paired-end reads as previously described (Wohlbach *et al.* 2014; Clowers *et al.* 2015). Briefly, paired-end reads were aligned to the assembly using Bowtie2 (Langmead and Salzberg 2012) with default settings with the exception of *–N=1* to allow for one mismatch. SNPs and indels were identified with GATK using base quality score recalibration, indel realignment, duplicate removal, and depth of coverage analysis (McKenna *et al.* 2010; Van der Auwera *et al.* 2013) using the default parameters with the exception for *-mbq 30* for the UnifiedGenotyper program. Variants were filtered using the following suggested GATK criteria: *QD< 2*, *FS > 60*, and *MQ< 40* for SNPs and *QD < 2*, and *FS > 200* for indels. The predicted SNPs

and indels were visually verified using IGV (Robinson *et al.* 2011; Thorvaldsdóttir *et al.* 2013), and the corrections were incorporated into the final assembly.

Preliminary genome annotation: The final assembly was annotated using YGAP (Proux-Wéra *et al.* 2012) and Liftover using S288C-R64 as the reference with *minIdentity 98%* and *minScore 100* (Kuhn *et al.* 2013). Initial annotations and quality checks for these methods proceeded separately until the annotations were integrated as noted below. We used BLASTN to find the *ScTAL1*, *CpXylA*, *PsXYL3*, and Kan^R genes from the xylose utilization cassette engineered into Y22-3 (Parreiras *et al.* 2014). Although we annotated these genes and examined their expression, we excluded them from all analyses of novel genes and non-syntenic homologs because they were not in the parent strain NRRL YB-210. All annotations with Ns within their sequences were removed. For protein-coding annotations, we determined whether the predicted open reading frame (ORF) would produce a valid protein by noting whether each ORF included:

1) A correct start codon
2) A correct stop codon
3) A sequence length divisible by 3
4) No internal stop codons

For both YGAP and Liftover annotations, we found that some predicted ORFs failed these tests. We considered any ORF annotations invalid if they did not pass all four criteria. We attempted to fix the annotations that lacked introns in the following ways:

Invalid start codons: For sequences containing only an invalid start codon, we searched upstream in frame until we encountered a valid start codon. If a stop codon was first encountered while searching upstream, we then searched downstream in frame for the next valid start codon.

Invalid stop codons or ORFs not divisible by 3: For sequences containing only an invalid stop codon, we searched downstream in frame until a valid stop codon was found. If the sequence had a start codon but was not divisible by 3, we removed the remainder (i.e. one or two bp) from the end of the annotation and attempted to find a downstream stop codon in the frame of the start codon.

Invalid start and stop codons: For sequences containing both an invalid start and stop codon, we first tried to fix the start codon as described above and, if successful, then attempted to fix the stop codon.

Invalid annotations due to internal stop codons were not fixed, nor were annotations that failed for other reasons not listed above. After attempting to fix the ORF annotations, we annotated all remaining invalid ORF annotations as putative pseudogenes.

Integrating YGAP and Liftover annotations: To integrate YGAP and Liftover gene annotations, we first compared the genomic coordinates. We categorized annotations as 1) agreeing in both the genomic coordinates and the annotation name, 2) agreeing in coordinates but disagreeing in name, 3) conflicting based upon their coordinates, or 4) conflicting and overlapping with multiple annotations. We also compared the coding sequences between introns and observed some that agreed,

others that conflicted, and others that conflicted with multiple annotations. We resolved conflicts using the following rules:

1) Keep the annotation that encodes a valid protein.
2) If both encode valid proteins, keep the annotation from Liftover.
3) If multiple Liftover annotations overlap (e.g. S288c pseudogenes or split ORFs), keep the annotation that matches a valid YGAP annotation.
4) For cases where multiple annotations conflict, keep only the annotations where YGAP and Liftover agree.

We also manually fixed some annotations as follows: The *YJM-GNAT* (Wei *et al.* 2007) gene was found in the sequence using BLASTN. ORFs *YMR084W* and *YMR085W* were merged into a complete opening reading frame, which is known to exist in some strains of *S. cerevisiae* (Kellis *et al.* 2003); since it is a paralog of *GFA1*, we called this merged annotation *GFA2* in Y22-3. Similarly, *YAR073W* and *YMR075W* comprise the complete ORF for *IMD1* (Escobar-Henriques and Daignan-Fornier 2001) in Y22-3. A paralog of *VTH1* was found by extending *YCR101C*, and the overlapping *YCR100C* and *YCR099C* ORFs were deleted. Finally, annotations to other non-S288c genes previously described (*MPR1*, *RTM1*, *BIO1*, *BIO6*, and *KHR1*) were renamed to match the literature (Borneman and Pretorius 2015).

We included other annotations found by Liftover (e.g. rRNAs, repeat_regions) within the final annotation. For tRNAs, we used both the annotations found by Liftover and YGAP, keeping the Liftover annotation in cases of disagreements regarding the genomic coordinates. Some centromeres were identified manually using BLAST. After manual inspection and circularization to match the orientation of S288c, the 2-micron

plasmid was annotated using Liftover (*S288C reference, minIdentity 85%, minScore 30*). The mitochondrial genome sequence was initially annotated using Liftover and finished manually using Geneious v. R6 (Kearse *et al.* 2012). Repeats were found using RepeatMasker v4.0.5, database 20140131 (Smit *et al.* 2013) with parameters *-species 'Saccharomyces cerevisiae' -s -no_is -cutoff 255 -frag 20000*.

Y22-3 RNA-Sequencing for gene expression analysis (UWBC dataset): Y22-3 was cultured as described previously (Parreiras *et al.* 2014). In brief, Y22-3 was inoculated to an optical cell density at 600 nm (OD_{600}) of 0.1 in 2 L Applikon bioreactors containing 1.8 L 10 g/L yeast extract, 20 g/L peptone, 60 g/L dextrose, 30 g/L xylose, 50 mM potassium phosphate, pH 5.0 (YPDX) and sparged with air. Cell samples were harvested during four stages of growth: minimal amount of glucose left (Residual Glucose Phase), during the transition from glucose to xylose consumption (Transition Phase), 1-10 hours after the transition (Early Xylose Phase), and ~20 hours after the transition (Xylose Phase) (Parreiras *et al.* 2014). For each harvest time point, 40 mL of culture were removed from the bioreactors; added to 5 mL ice-cold 95:5 ethanol:water-saturated phenol, pH 6.6; and mixed by inversion. Fixed cells were then pelleted by centrifugation at 10,000 RCF for 5 min at 4 ^{o}C. The supernatant was decanted and the remaining pellet flash frozen in a dry ice-ethanol bath. Total RNA was extracted from the Y22-3 cell pellet by hot phenol extraction (Gasch 2002). RNA was further purified using RNeasy Plus columns (Qiagen) according to manufacturer's protocol.

Total RNA was submitted to the UWBC Gene Expression Center, which verified purity and integrity using a NanoDrop2000 Spectrophotometer and an Agilent 2100 BioAnalyzer, respectively. Samples that met the Illumina sample input guidelines were

prepared according the TruSeq® Stranded Total RNA Sample Preparation Guide (Rev. E) using the Illumina® TruSeq® Stranded Total RNA Sample Preparation kits (Illumina Inc., San Diego, California, USA) with minor modifications. For each library preparation, 2 µg of total RNA was subjected to ribosomal RNA depletion using the EpiCentre RiboZero™ Gold Ribosomal RNA Removal (Yeast) kit (EpiCentre Inc., Madison, WI, USA) as directed. Ribosomal RNA-depleted RNA samples were purified using paramagnetic beads (Agencourt RNA Clean XP beads, Beckman Coulter, Indianapolis IN, USA). Subsequently, each rRNA-depleted sample was fragmented using divalent cations under elevated temperature. The fragmented RNA was synthesized into double-stranded cDNA using SuperScript II Reverse Transcriptase (Invitrogen, Carlsbad, California, USA) and random primers for first-strand cDNA synthesis, followed by second-strand synthesis using DNA Polymerase I, and RNAse H to degrade the mRNA. Double-stranded cDNA was purified using paramagnetic beads (Agencourt AMPure XP beads, Beckman Coulter). The cDNA products were incubated with Klenow DNA Polymerase to add an ‘A’ nucleotide (deoxyadenosine monophosphate) to the 3’ end of the blunt DNA fragments. DNA fragments were ligated to forked Illumina adapters, which have a single ‘T’ nucleotide (deoxythymidine monophosphate) overhang at their 3’ end. The adapter-ligated DNA products were purified using paramagnetic beads. Adapter-ligated DNA was amplified in a Linker Mediated PCR reaction (LM-PCR) for 11 cycles using Phusion™ DNA Polymerase and Illumina's PE genomic DNA primer set, followed by purification with paramagnetic beads. Quality and quantity of the finished libraries were assessed using an Agilent DNA1000 chip (Agilent Technologies, Inc., Santa Clara, CA, USA) and Qubit® dsDNA HS Assay Kit (Invitrogen, Carlsbad,

California, USA), respectively. Cluster generation was performed using standard Cluster Kits (v3) and the Illumina Cluster Station. Single-end, 100-bp sequencing was performed using standard SBS chemistry (v3) on an Illumina HiSeq 2500 sequencer. Images were analyzed using the standard Illumina Pipeline, version 1.8.2.

RNA-Sequencing for de novo transcriptome assembly (JGI dataset): Clones derived from Y22-3 were inoculated to an optical cell density at 600 nm (OD_{600}) of 0.1 in 2 L Applikon bioreactors containing 1.8 L YPDX or AFEX-pretreated corn stover hydrolysate and sparged with air (aerobic) or nitrogen (anaerobic). Cell samples were harvested during six stages of growth: during glucose consumption (Late Glucose Phase), minimal amount of glucose left (Residual Glucose Phase), during the transition from glucose to xylose consumption (Transition Phase), 2-6 hours after the transition (Early Xylose Phase), 8 hours after transition to xylose (Intermediate Xylose Phase) and ~20 hours after the transition (Xylose Phase) (Parreiras *et al.* 2014). RNA was purified as described above and submitted to JGI. Plate-based RNA sample preparation was performed on the PerkinElmer Sciclone NGS robotic liquid handling system using Illumina's TruSeq Stranded mRNA HT sample prep kit (http://support.illumina.com/sequencing/sequencing_kits/truseq_stranded_mrna_ht_sample_prep_kit.html) following the protocol outlined by Illumina in their user guide and with the following conditions: total RNA starting material was 1 µg per sample, and 10 cycles of PCR were used for library amplification.

The library was quantified using KAPA Biosystem's next-generation sequencing library qPCR kit and run on a Roche LightCycler 480 real-time PCR instrument. The quantified library was then multiplexed with other libraries for a pool size of six, and the

pool was then prepared for sequencing on the Illumina HiSeq sequencing platform utilizing a TruSeq paired-end cluster kit v3 and Illumina's cBot instrument to generate a clustered flowcell for sequencing. Sequencing of the flowcell was performed on the Illumina HiSeq 2000 sequencer using a TruSeq SBS sequencing kit 200 cycles v3 following a 2x150 indexed run recipe.

Proteomic experiments and validation (Protein Method): Proteomic experiments were performed similarly to our previous approach (Hebert *et al.* 2014). For protein extraction and digestion, yeast cell pellets were lysed by glass bead milling (Retsch GmbH, Germany). Lysate protein concentration was measured via bicinchoninic acid protein assay (Thermo Pierce, Rockford, IL), and yeast proteins were reduced through incubation in 5 mM dithiothreitol for 45 min at 58 °C. Free cysteines were alkylated in 15 mM iodoacetamide in the dark for 30 min. The alkylation was stopped with 5 mM DTT. A 1 mg protein aliquot was digested overnight at room temperature in 1.5 M Urea with trypsin (Promega, Madison, WI) added at a 1:50 (w/w) enzyme to protein ratio. Digestions were quenched by the addition of trifluoroacetic acid and were then desalted over tC18 Sep-Pak cartridges (Waters, Milford, MA).

For online nanoflow liquid chromatography tandem mass spectrometry (nLC-MS/MS), reversed phase columns were packed in-house using 75 µm ID, 360 µm OD bare fused silica capillary. A nanoelectrospray tip was laser pulled (Sutter Instrument Company, Novato, CA) and packed with 1.7 µm diameter, 130 Å pore size Ethylene Bridged Hybrid C18 particles (Waters) to a length of 30-35 cm. Buffer A consisted of 0.2% formic acid and 5% DMSO in water, and Buffer B consisted of 0.2% formic acid in acetonitrile. Two µg of peptides were loaded onto the column in 95% buffer A for 12 min

at 300 min^{-1}. Gradient elution was performed at 300 nL min^{-1}, and gradients increased linearly from 5 to 35% buffer B over 190 min, followed by an increase to 70% B at 215 min and a wash at 70% B for 5 min. The column was then re-equilibrated at 5% B for 20 min. Eluting peptides were ionized with electrospray ionization at +2 kV, and the inlet capillary temperature was held at 300 °C on an ion-trap Orbitrap hybrid mass spectrometer (Orbitrap Elite, Thermo Fisher Scientific, San Jose, CA). Survey scans of peptide precursors were collected over the 300-1500 Thompson range in the Orbitrap with an automatic gain control target value of 1,000,000 (50 ms maximum injection time), followed by data-dependent ion trap MS/MS scans using collisional activation dissociation (CAD) of the 20 most intense peaks (AGC target value of 5,000 and maximum injection times of 100 ms). Precursors with charge states equal to one or unassigned were rejected.

A total of three replicates were performed, two of which were biological (i.e. independent cultures) and one of which was technical (i.e. a second pellet was collected for one of the biological replicates). These three pellets were collected separately and also handled separately at each step of the proteomics analysis (including cell lysis, proteolytic digestion, and LC-MS/MS analysis). Peptide mixtures from each of these pellets were injected/analyzed once, giving a total of three replicates. Since peptide quantification was highly correlated for both the biological and technical replicates ($R^2 > 0.96$), we processed all data in batch but used the match-between-runs feature of MaxQuant (Version 1.4.1.2) (Cox and Mann 2008), thus allowing validation of the expression of predicted proteins by any of the three replicates.

Raw data was processed using MaxQuant (Version 1.4.1.2) (Cox and Mann 2008), and tandem mass spectra were searched with the Andromeda search algorithm (Cox *et al.* 2011). Oxidation of methionine was specified as a variable modification, while carbamidomethylation of cysteine was a set as a fixed modification. A precursor search tolerance of 20 ppm and a product mass tolerance of 0.35 Da were used for searches, and three missed cleavages were allowed for full trypsin specificity. Peptide spectral matches (PSMs) were made against a target-decoy custom database of the Y22-3 protein-coding annotations (created August 25, 2015), which was concatenated with a reversed sequence version of the forward database. Peptides were filtered to a 1% false discovery rate (FDR), and a 1% protein FDR was applied according to the target-decoy method. Proteins were identified using at least one peptide (razor + unique), where the razor peptide is defined as a non-unique peptide assigned to the protein group with the most other peptides (Occam's razor principle). Using these methods, we identified 29,227 unique peptides (**Table S4**). For the final analysis, we only considered predicted proteins to be validated we could detect at least one unique peptide that could be unambiguously assigned to it.

Validating genes by RNA-Sequencing (FPKM Method): For validation of predicted protein-coding genes by the FPKM (Fragments Per Kilobase of transcript per Million mapped reads) Method, we first trimmed the aligned the UWBC single-end Illumina data using Trimmomatic (Bolger *et al.* 2014) with the following rules: 1) remove any number of bp from 3’ end that have the average quality score < 26 in a 3-bp sliding window, and 2) keep the trimmed read if 25 or more bp are left. The trimmed reads were then aligned using Tophat v2.1.0 (Trapnell *et al.* 2012). We then removed non-

uniquely mapped reads. Finally, we extracted counts and calculated the FPKM for each gene annotation using Cufflinks v2.2.1 (Trapnell *et al.* 2012).

De novo transcriptome assembly (Transcriptome Method): To describe transcriptional activity of Y22-3 over a wide range of conditions, nearly 1.5 billion (1,433,309,474) paired-end Illumina RNA-Seq reads from the JGI dataset were assembled de novo to yield a generalized transcriptome model (Parreiras *et al.* 2014). To remove both low-quality and nucleotide composition-biased parts of the sequencing reads, the Trimmomatic software (Bolger *et al.* 2014) was applied to pre-process the reads with the following rules: 1) remove the first 12 bp from 5' end, 2) remove any number of bp from 3' end that have the average quality score < 30 in a 3-bp sliding window, and 3) keep the trimmed read if 36 or more bp are left.

Transcriptome assembly was performed using the Trinity pipeline (Grabherr *et al.* 2011). The pool of reads was normalized to a target coverage of 50 using Trinity's in silico normalization routine. Transcriptome assembly with default parameters produced numerous artificial fusion transcripts. To optimize the assembly parameters for our particular case, extensive parameter scanning and optimization were performed by generating 270 de novo assemblies that combined 10 levels of *minimal k-mer coverage* (Inchworm stage of the Trinity), three levels of *minimal glue*, three levels of *minimal iso ratio*, and three levels of *glue factor* (Chrysalis stage). Optimization of the Butterfly-stage parameters was not recommended by the Trinity developers.

Selection of the best assembly was performed with the aid of the DETONATE package (Li *et al.* 2014), using both the RSEM-EVAL and REF-EVAL pipelines. Our

preliminary applications of DETONATE to smaller-size assemblies, combined with visual assessment of the assembly quality after subsequent mapping to the genome sequence, revealed that 3 out of 47 output statistics generated by the package - *Transcript Length Distribution Related Factors* (when maximized), *Unweighted K-mer KL_A_to_M* (when minimized), and *Unweighted_Pair_F1* (when maximized) - are better representatives of the overall assembly quality for our transcriptome. We selected the three candidate assemblies with top scores according to each of the three statistics. The final assembly was selected by minimizing the sum of ranks; this assembly belonged to the top 5% of assemblies for all three ranked lists. The following advanced Trinity parameters were used to generate the optimized transcriptome assembly: *min_kmer_cov 32* (Inchworm stage), *min_glue 4*, *min_iso_ratio 0.01*, and *glue_factor 0.01* (Chrysalis stage). The transcripts from the optimized transcriptome assembly were mapped onto the Y22-3 genome sequence via the first stage of the PASA pipeline (Haas *et al.* 2003) with blat and gmap aligners and the following options: --*MAX_INTRON_LENGTH 2000* and *--transcribed_is_aligned_orient*. Alignment stringency was set to minimum of 95% identity across at least 90% of the transcript sequence. Visual comparison of the mapping results derived from the optimized and default-parameter assemblies (**Figure S1**) revealed that cases of artificial both-strand coverage by predicted transcripts, which were abundant in the default assembly, were essentially eliminated in the optimized assembly, without sacrificing sensitivity (seen as coverage of the genomic features predicted at DNA level).

Comparing strains by presence of genes using TBLASTN: To compare the genome content of several diverse *S. cerevisiae* genomes, we developed a Novelty Metric to integrate TBLASTN results. We used a TBLASTN word size of two. From the bits for the best TBLASTN hit of the query gene against a given subject strain, we subtracted the number of bits for the best hit against S288c. We then divided that value by the number of bits for the best hit from all of the strains searched for that particular gene. Negative values were set to zero, and the result was scaled from 0-100 (**Equation 1**).

$$MatchScore(gene_i, strain_j) = \max[0, \frac{Bits(gene_i, strain_j) - Bits(gene_i, strain_{S288c})}{\max(1e{-}300, \max_{k \in strain}(Bits(gene_i, strain_k) - Bits(gene_i, strain_{S288c})))}] \times 100$$

Equation 1

This Novelty Metric is capable of distinguishing novel genes, even when there is a relatively close homolog in S288c. We generated heat maps of previously characterized non-S288c genes (Goto *et al.* 1991; Borneman and Pretorius 2015; Kowalec *et al.* 2015) and for genes predicted but not characterized in other strains (**Figure 3**). We excluded *IMI1* from strain W303 because the difference in gene content is caused by the absence of a stop codon relative to S288c, which fuses two adjacent genes and their intergenic region (Kowalec *et al.* 2015), rendering the Novelty Metric uninformative. We recorded the value as "not applicable" for any query gene where the Novelty Metric for the all strains, including the source strain, was zero (e.g. the S288c hit was equally good, or the novel ORF predicted by other authors did not match the published genome sequence).

Examination of non-S288c genes with predicted functions: Of the 92 genes predicted in Y22-3 but not in S288c we carefully examined those genes where we could assign a putative function, leading to the 43 genes in **Figure 4** and **Table S6**. These 43 genes exclude repeat sequences missed by RepeatMasker (e.g. some Y' helicases) and genes homologous to genes with no known functions (e.g. *PAU* (which encode seripauperins) and *COS* genes) (**Table S7**). *KHR1* (Goto *et al.* 1991) was retained for these analyses, even though it was found within a LTR region. For each of these genes, we used the WU-BLASTP web tool on SGD (with default settings, except with no filters) to recover and record the best High-scoring Segment Pair (HSP) in S288c. We manually examined other close matches and gene annotations in SGD. We used the BLASTP web tool on NCBI (with default settings, except with no filters) to search the "nr" GenBank database for annotated proteins in other organisms, including other strains of *S. cerevisiae*. We manually examined entries for other close matches to determine whether putative functional inferences could be made or whether similar genes had already been named. For each of the 28 novel genes named, the best hit in S288c was already annotated as orthologous in the Y22-3 genome (i.e. it was not a reciprocal best BLAST hit). This criterion excluded 3 clusters of subtelomeric genes that may represent translocations. Furthermore, except for systematic names, each of these genes did not closely match a non-S288c gene that had already been named in another strain of *S. cerevisiae*.

Phylogenetic comparison to other *Saccharomyces* strains: To study the relationship between Y22-3 and *Saccharomyces* strains, we generated a maximum likelihood (ML) phylogeny using RAxML v. 8.1.20 (Stamatakis 2014) on a nucleotide

dataset containing orthologous protein-coding sequences conserved across all strains, including the outgroup *S. paradoxus* (Scannell *et al.* 2011). We performed multiple sequence alignments using DIALIGN2 (Morgenstern 1999) and removed indels, ambiguous nucleotides, and heterozygous nucleotides by codon as previously described (Hittinger *et al.* 2010). We reconstructed the ML phylogeny using 30 BFGS searches and the GTR+GAMMA (Yang 1993) model of nucleotide evolution. Bootstrap support values were determined for the best ML tree using 100 pseudoreplicates.

R Software Used: In house scripts for data analysis and plotting were written in R (R Core Team 2015) using the packages *Biostrings* (Pages *et al.*), *VennDiagram* (Chen and Boutros 2011), gplots (Warnes *et al.* 2015), *ggtree* (Yu *et al.*), and *ggplot2* (Wickham 2009).

LITERATURE CITED

Van der Auwera, G. A., M. O. Carneiro, C. Hartl, R. Poplin, G. Del Angel *et al.*, 2013 From FastQ Data to High-Confidence Variant Calls: The Genome Analysis Toolkit Best Practices Pipeline, pp. 11.10.1–11.10.33 in *Current Protocols in Bioinformatics*, edited by A. Bateman, W. R. Pearson, L. D. Stein, G. D. Stormo, and J. R. Yates. John Wiley & Sons, Inc., Hoboken, NJ, USA.

Baker, E., B. Wang, N. Bellora, D. Peris, A. B. Hulfachor *et al.*, 2015 The genome sequence of *Saccharomyces eubayanus* and the domestication of lager-brewing yeasts. Mol Biol Evol 32: 2818–31.

Bankevich, A., S. Nurk, D. Antipov, A. A. Gurevich, M. Dvorkin *et al.*, 2012 SPAdes: A

New Genome Assembly Algorithm and Its Applications to Single-Cell Sequencing. J Comput Biol 19: 455–477.

Bolger, A. M., M. Lohse, and B. Usadel, 2014 Trimmomatic: A flexible trimmer for Illumina Sequence Data. Bioinformatics 30: 2114–2120.

Bonfield, J. K., and A. Whitwham, 2010 Gap5—editing the billion fragment sequence assembly. Bioinformatics 26: 1699–1703.

Borneman, A. R. R., and I. S. S. Pretorius, 2015 Genomic Insights into the *Saccharomyces* sensu stricto Complex. Genetics 199: 281–291.

Chen, H., and P. C. Boutros, 2011 VennDiagram: a package for the generation of highly-customizable Venn and Euler diagrams in R. BMC Bioinformatics 12: 1–7.

Chin, C.-S., D. H. Alexander, P. Marks, A. A. Klammer, J. Drake *et al.*, 2013 Nonhybrid, finished microbial genome assemblies from long-read SMRT sequencing data. Nat Meth 10: 563–569.

Clowers, K. J., J. Heilberger, J. S. Piotrowski, J. L. Will, and A. P. Gasch, 2015 Ecological and Genetic Barriers Differentiate Natural Populations of *Saccharomyces cerevisiae*. Mol Biol Evol 32: 2317–2327.

Cox, J. J., and M. Mann, 2008 MaxQuant enables high peptide identification rates, individualized p.p.b.-range mass accuracies and proteome-wide protein quantification. Nat Biotech 26: 1367–1372.

Cox, J., N. Neuhauser, A. Michalski, R. A. Scheltema, J. V Olsen *et al.*, 2011 Andromeda: A Peptide Search Engine Integrated into the MaxQuant Environment.

J Proteome Res 10: 1794–1805.

English, A. C., S. Richards, Y. Han, M. Wang, V. Vee *et al.*, 2012 Mind the Gap: Upgrading Genomes with Pacific Biosciences RS Long-Read Sequencing Technology. PLoS One 7: e47768.

Escobar-Henriques, M., and B. Daignan-Fornier, 2001 Transcriptional Regulation of the Yeast GMP Synthesis Pathway by Its End Products. J Biol Chem 276: 1523–1530.

Gasch, A. P., 2002 Yeast genomic expression studies using DNA microarrays. Methods Enzymol 350: 393–414.

Goto, K., H. Fukuda, K. Kichise, K. Kitano, and S. Hara, 1991 Cloning and nucleotide sequence of the *KHS* killer gene of *Saccharomyces cerevisiae*. Agric Biol Chem 55: 1953–8.

Grabherr, M. G., B. J. Haas, M. Yassour, J. Z. Levin, D. A. Thompson *et al.*, 2011 Full-length transcriptome assembly from RNA-Seq data without a reference genome. Nat Biotech 29: 644–652.

Gurevich, A., V. Saveliev, N. Vyahhi, and G. Tesler, 2013 QUAST: quality assessment tool for genome assemblies. Bioinformatics 29: 1072–5.

Haas, B. J., A. L. Delcher, S. M. Mount, J. R. Wortman, R. K. Smith Jr *et al.*, 2003 Improving the *Arabidopsis* genome annotation using maximal transcript alignment assemblies. Nucleic Acids Res 31: 5654–5666.

Hebert, A. S., A. L. Richards, D. J. Bailey, A. Ulbrich, E. E. Coughlin *et al.*, 2014 The One Hour Yeast Proteome. Mol Cell Proteomics 13: 339–347.

Heo, Y., X.-L. Wu, D. Chen, J. Ma, and W.-M. Hwu, 2014 BLESS: Bloom filter-based error correction solution for high-throughput sequencing reads. Bioinformatics 30: 1354–1362.

Hittinger, C. T., M. Johnston, J. T. Tossberg, and A. Rokas, 2010 Leveraging skewed transcript abundance by RNA-Seq to increase the genomic depth of the tree of life. Proc Natl Acad Sci U S A 107: 1476–1481.

Ilie, L., and M. Molnar, 2013 RACER: Rapid and accurate correction of errors in reads. Bioinformatics 29: 2490–2493.

Imai, T., 2013 Sprai = single pass read accuracy improver <http://zombie.cb.k.u-tokyo.ac.jp/sprai/index.html>.

Kamada, M., S. Hase, K. Sato, A. Toyoda, A. Fujiyama *et al.*, 2014 Whole Genome Complete Resequencing of *Bacillus subtilis* Natto by Combining Long Reads with High-Quality Short Reads. PLoS One 9: e109999.

Kearse, M., R. Moir, A. Wilson, S. Stones-Havas, M. Cheung *et al.*, 2012 Geneious Basic: An integrated and extendable desktop software platform for the organization and analysis of sequence data. Bioinformatics 28: 1647–1649.

Kellis, M., N. Patterson, M. Endrizzi, B. Birren, and E. S. Lander, 2003 Sequencing and comparison of yeast species to identify genes and regulatory elements. Nature 423: 241–254.

Koren, S., G. P. Harhay, T. P. L. Smith, J. L. Bono, D. M. Harhay *et al.*, 2013 Reducing assembly complexity of microbial genomes with single-molecule sequencing. Genome Biol 14: R101.

Kowalec, P., M. Grynberg, B. Pająk, A. Socha, K. Winiarska *et al.*, 2015 Newly identified protein Imi1 affects mitochondrial integrity and glutathione homeostasis in *Saccharomyces cerevisiae*. FEMS Yeast Res 15: fov048.

Krumsiek, J., R. Arnold, and T. Rattei, 2007 Gepard: a rapid and sensitive tool for creating dotplots on genome scale. Bioinformatics 23: 1026–1028.

Kuhn, R. M., D. Haussler, and W. J. Kent, 2013 The UCSC genome browser and associated tools. Brief Bioinform 14: 144–161.

Langmead, B., and S. L. Salzberg, 2012 Fast gapped-read alignment with Bowtie 2. Nat Meth 9: 357–359.

Li, B., N. Fillmore, Y. Bai, M. Collins, J. A. Thomson *et al.*, 2014 Evaluation of de novo transcriptome assemblies from RNA-Seq data. Genome Biol 15: 553.

McKenna, A., M. Hanna, E. Banks, A. Sivachenko, K. Cibulskis *et al.*, 2010 The Genome Analysis Toolkit: A MapReduce framework for analyzing next-generation DNA sequencing data. Genome Res 20: 1297–1303.

Morgenstern, B., 1999 DIALIGN 2: improvement of the segment-to-segment approach to multiple sequence alignment. Bioinformatics 15: 211–218.

Myers, E. W., G. G. Sutton, A. L. Delcher, I. M. Dew, D. P. Fasulo *et al.*, 2000 A whole-genome assembly of *Drosophila*. Science 287: 2196–2204.

Pages, H., P. Aboyoun, R. Gentleman, and S. DebRoy Biostrings: String objects representing biological sequences, and matching algorithms.

Parreiras, L. S., R. J. Breuer, R. Avanasi Narasimhan, A. J. Higbee, A. La Reau *et al.*,

2014 Engineering and Two-Stage Evolution of a Lignocellulosic Hydrolysate-Tolerant *Saccharomyces cerevisiae* Strain for Anaerobic Fermentation of Xylose from AFEX Pretreated Corn Stover. PLoS One 9: e107499.

Proux-Wéra, E., D. Armisén, K. P. Byrne, K. H. Wolfe, E. Proux-Wera *et al.*, 2012 A pipeline for automated annotation of yeast genome sequences by a conserved-synteny approach. BMC Bioinformatics 13: 237.

R Core Team, 2015 R: A Language and Environment for Statistical Computing.

Robinson, J. T., H. Thorvaldsdottir, W. Winckler, M. Guttman, E. S. Lander *et al.*, 2011 Integrative genomics viewer. Nat Biotech 29: 24–26.

Scannell, D. R., O. A. Zill, A. Rokas, C. Payen, M. J. Dunham *et al.*, 2011 The awesome power of yeast evolutionary genetics: new genome sequences and strain resources for the *Saccharomyces* sensu stricto genus. G3 (Bethesda) 1: 11–25.

Smit, A. F. A., R. Hubley, and P. Green, 2013 RepeatMasker Open-4.0 <http://www.repeatmasker.org>.

Stamatakis, A., 2014 RAxML version 8: a tool for phylogenetic analysis and post-analysis of large phylogenies. Bioinformatics 30: 1312–1313.

Thorvaldsdóttir, H., J. T. Robinson, and J. P. Mesirov, 2013 Integrative Genomics Viewer (IGV): high-performance genomics data visualization and exploration. Brief Bioinform 14: 178–192.

Trapnell, C., A. Roberts, L. Goff, G. Pertea, D. Kim *et al.*, 2012 Differential gene and transcript expression analysis of RNA-seq experiments with TopHat and Cufflinks.

Nat Protoc 7: 562–578.

Warnes, G. R., B. Bolker, L. Bonebakker, R. Gentleman, W. H. A. Liaw *et al.*, 2015 gplots: Various R programming tools for plotting data.

Wei, W., J. H. McCusker, R. W. Hyman, T. Jones, Y. Ning *et al.*, 2007 Genome sequencing and comparative analysis of *Saccharomyces cerevisiae* strain YJM789. Proc Natl Acad Sci 104: 12825–12830.

Wickham, H., 2009 *ggplot2: elegant graphics for data analysis*. Springer New York.

Wohlbach, D. J., N. Rovinskiy, J. a. Lewis, M. Sardi, W. S. Schackwitz *et al.*, 2014 Comparative genomics of *Saccharomyces cerevisiae* natural isolates for bioenergy production. Genome Biol Evol 6: 2557–2566.

Yang, Z., 1993 Maximum-likelihood estimation of phylogeny from DNA sequences when substitution rates differ over sites. Mol Biol Evol 10: 1396–1401.

Yu, G., D. Smith, H. Zhu, Y. Guan, and T. T.-Y. Lam ggtree: an R package for visualization and annotation of phylogenetic tree with different types of meta-data.

Zerbino, D. R., and E. Birney, 2008 Velvet: algorithms for de novo short read assembly using de Bruijn graphs. Genome Res 18: 821–829.
