# Supplementary material for: Genome Sequence and Analysis of a Stress-Tolerant, Wild-Derived Strain of Saccharomyces cerevisiae Used in Biofuels Research
Source: G3 (Bethesda). 2016 Apr 16;6(6):1757–66. doi: 10.1534/g3.116.029389 (PMC4889671; doi:10.1534/g3.116.029389)
Supplement: Supplemental Material [file supp_g3.116.029389_FileS2.pdf]

FILE S2: GENOME ASSEMBLY SCRIPTS

Sprai+Quiver

```
ezez_vx1.pl ec.spec pbasm.spec
pbalign.py --nproc 8 --forQuiver --tmpDir /tmp all.fofn $assembly $b.cmp.h5
quiver -j 8 $b.cmp.h5 -r $assembly -o consensus.fa -o variants.gff
```

```
ec.spec
#### common ####
# input_for_database: filtered subreads in fasta or fastq format
input_for_database PacBio/Y22-3.pacbio.filtered_subreads.fastq

# min_len_for_query: the subreads longer than or equal to this value will be corrected
min_len_for_query 500

#if you don't know the estimated genome size, give a large number
estimated_genome_size 12156677
#if you don't know the estimated depth of coverage, give 0
estimated_depth 100

#
ca_path Programs/wgs-8.0/Linux-amd64/bin/

# the number of processes used by all vs. all alignment
# = 'partition' (in single node mode)
# = 'pre_partition' * 'partition' (in many node mode)
pre_partition 2
partition 12

# sprai prefer full paths
# if you use ezez4qsub*.pl. you MUST specify blast_path & sprai_path
# blast_path: where blastn and makeblastdb exist in
blast_path /usr/bin/blastn
# sprai_path: where binaries of sprai (bfmt72s, nss2v_v3 and so on) exist in
sprai_path /home/smcilwain/bin/

#### many node mode (advanced) ####

#sge: options for all the SGE jobs
#sge -soft -l ljob,lmem,sjob
#queue_req: additional options for all the SGE jobs
#queue_req -l s_vmem=4G -l mem_req=4
#longestXx_queue_req: if valid, displaces queue_req
#longestXx_queue_req -l s_vmem=64G -l mem_req=64
```

#BLAST_RREQ: additional options for SGE jobs of all vs. all alignment
#BLAST_RREQ -pe def_slot 4

common (advanced)

used by blastn
word_size 18
evalue 1e-50
num_threads 1

#valid_voters 11

#trim: both ends of each alignment by blastn will be trimmed 'trim' bases to detect chimeric reads
trim 42

pbasm.spec

Copyright (c) 2011-2013, Pacific Biosciences of California, Inc.
#
All rights reserved.
#
Redistribution and use in source and binary forms, with or without
modification, are permitted (subject to the limitations in the
disclaimer below) provided that the following conditions are met:
#
* Redistributions of source code must retain the above copyright
notice, this list of conditions and the following disclaimer.
#
* Redistributions in binary form must reproduce the above
copyright notice, this list of conditions and the following
disclaimer in the documentation and/or other materials provided
with the distribution.
#
* Neither the name of Pacific Biosciences nor the names of its
contributors may be used to endorse or promote products derived
from this software without specific prior written permission.
#
NO EXPRESS OR IMPLIED LICENSES TO ANY PARTY'S PATENT RIGHTS ARE
GRANTED BY THIS LICENSE. THIS SOFTWARE IS PROVIDED BY PACIFIC
BIOSCIENCES AND ITS CONTRIBUTORS "AS IS" AND ANY EXPRESS OR
IMPLIED
WARRANTIES, INCLUDING, BUT NOT LIMITED TO, THE IMPLIED WARRANTIES
OF MERCHANTABILITY AND FITNESS FOR A PARTICULAR PURPOSE ARE
DISCLAIMED. IN NO EVENT SHALL PACIFIC BIOSCIENCES OR ITS
CONTRIBUTORS BE LIABLE FOR ANY DIRECT, INDIRECT, INCIDENTAL,
SPECIAL, EXEMPLARY, OR CONSEQUENTIAL DAMAGES (INCLUDING, BUT NOT

```
# LIMITED TO, PROCUREMENT OF SUBSTITUTE GOODS OR SERVICES; LOSS
OF
# USE, DATA, OR PROFITS; OR BUSINESS INTERRUPTION) HOWEVER CAUSED
AND
# ON ANY THEORY OF LIABILITY, WHETHER IN CONTRACT, STRICT LIABILITY,
# OR TORT (INCLUDING NEGLIGENCE OR OTHERWISE) ARISING IN ANY WAY
OUT
# OF THE USE OF THIS SOFTWARE, EVEN IF ADVISED OF THE POSSIBILITY OF
# SUCH DAMAGE.
#
```


```
unitigger = bogart
#utgErrorRate = 0.015
#utgErrorLimit = 4.5

cnsErrorRate = 0.25
cgwErrorRate = 0.25
ovlErrorRate = 0.015

frgMinLen = 1000
ovlMinLen = 40

merSize=14

merylMemory = 16384
merylThreads = 8

ovlStoreMemory = 16384

# grid info
useGrid = 0
scriptOnGrid = 0
frgCorrOnGrid = 0
ovlCorrOnGrid = 0

sge = -S /bin/bash -V -q all.q
#sge = -S /bin/bash -sync y -V -q all.q
sgeScript = -pe threads 1
sgeConsensus = -pe threads 1
sgeOverlap = -pe threads 4
sgeFragmentCorrection = -pe threads 4
sgeOverlapCorrection = -pe threads 1

#ovlHashBits = 22
#ovlHashBlockLength = 46871347
#ovlRefBlockSize =  537
```

ovlHashBits = 25
ovlThreads = 4
ovlHashBlockLength = 50000000
ovlRefBlockSize = 100000000

ovlConcurrency = 6
frgCorrThreads = 4
frgCorrBatchSize = 100000
ovlCorrBatchSize = 100000

cnsMinFrags = 7500
cnsConcurrency = 24

# change sgeName every time if you do not want to wait for the jobs not necessary to wait
sgeName = iroha

PBCR

PBcR -length 500 -partitions 200 -l Y22-3.pacbio -s pacbio.spec -fastq $pacbio_fastq genomeSize=12156677

pacbio.spec
merSize=14

PBCR+Illumina

pacbio_fastq="Y22-3.pacbio.filtered_subreads.fastq"

pbcr_length=500
pbcr_partions=200
genome_size=12156677

illumina_fastq_R1=subsample_R1.fastq
illumina_fastq_R2=subsample_R2.fastq

illumina_insert_size=1000
illumina_insert_size_d=50

```
if [ ! -e illumnia.frg ]; then
  echo "creating illumina.frg"
  cmd="fastqToCA -libraryname illumina -technology illumina -type sanger \
    -insertsize $illumina_insert_size $illumina_insert_size_d \
    -innie -mates $illumina_fastq_R1,$illumina_fastq_R2"
```

```
  echo $cmd
  $cmd > illumina.frg.tmp
  mv illumina.frg.tmp illumina.frg
  echo "illumina.frg created"
fi

#Run PBcR
PBcR -length 500 -partitions 200 -l Y22-3.pacbio.illumina -s pacbio.illumina.spec -fastq
$pacbio_fastq genomeSize=12156677 illumina.frg
```

HGAP

```
if [ ! -e hgap3 ]; then
  mkdir hgap3
fi

TMP=/home/GLBRCORG/smcilwain/tmp
SHARED_DIR=/home/GLBRCORG/smcilwain/shared_dir

echo "Running smrtpipe.py"

smrtpipe.py -DTMP=$TMP -DSHARED_DIR=$SHARED_DIR --params hgap3.xml --
output=hgap3 xml:my_inputs.xml >& hgap3.log
```

hgap3.xml:

```
<?xml version="1.0"?>
<smrtpipeSettings>
  <global>
    <param name="version">
        <value>3</value>
    </param>
    <param name="fetch">
        <value>common/protocols/preprocessing/Fetch.1.xml</value>
    </param>
    <param name="state">
        <value>active</value>
    </param>
    <param name="Control Filtering">
        <value>common/protocols/control/KeepControlReads.1.xml</value>
    </param>
    <param name="assembly">
        <value>common/protocols/assembly/PreAssemblerHGAP.3.xml</value>
    </param>
    <param name="consensus">
        <value>common/protocols/consensus/AssemblyPolishing.1.xml</value>
```

```
    </param>
    <param name="description">
      <value>(BETA) HGAP version 3. PacBio de novo assembler optimized for
speed.</value>
    </param>
    <param name="filtering">
      <value>common/protocols/filtering/PreAssemblerSFilter.1.xml</value>
    </param>
    <param name="mapping">
      <value>common/protocols/mapping/BLASR.1.xml</value>
    </param>
    <param name="otfReference">
      <value>reference</value>
    </param>
    <param name="referenceUploader">

<value>common/protocols/referenceuploader/ReferenceUploaderUnitig.1.xml</value>
    </param>
    <param name="deferRefCheck">
      <value>True</value>
    </param>
    <param name="name">
      <value>RS_HGAP_Assembly_3</value>
    </param>
  </global>
  <module name="P_Fetch">
    <param name="moduleName">
      <value>P_Fetch</value>
    </param>
    <param name="description">
      <value>Sets up inputs</value>
    </param>
  </module>
  <module name="P_Filter">
    <param name="moduleName">
      <value>P_Filter</value>
    </param>
    <param name="minSubReadLength">
      <value>500</value>
    </param>
    <param name="description">
      <value>Filter reads for use in the pre-assembly step of HGAP, the hierarchical
genome assembly process.</value>
    </param>
    <param name="minLength">
      <value>100</value>
```

```
    </param>
    <param name="readScore">
      <value>0.80</value>
    </param>
  </module>
  <module name="P_FilterReports">
    <param name="moduleName">
      <value>P_FilterReports</value>
    </param>
  </module>
  <module name="P_PreAssemblerDagcon">
    <param name="minLongReadLength">
      <value>1000</value>
    </param>
    <param name="totalBestn">
      <value>24</value>
    </param>
    <param name="minCorCov">
      <value>6</value>
    </param>
    <param name="moduleName">
      <value>P_PreAssemblerDagcon</value>
    </param>
    <param name="splitBestn">
      <value>10</value>
    </param>
    <param name="targetChunks">
      <value>6</value>
    </param>
    <param name="computeLengthCutoff">
      <value>True</value>
    </param>
    <param name="blasrOpts">
      <value>-noSplitSubreads -minReadLength 200 -maxScore -1000 -maxLCPLength
16</value>
    </param>
    <param name="title">
      <value>Using DAG-based consensus algorithm, pre-assemble long reads as the
first step of the Hierarchical Genome Assembly process (HGAP). Version 2 is a
stepping stone for scaling to much larger genomes.</value>
    </param>
  </module>
  <module name="P_AssembleUnitig">
    <param name="merSize">
      <value>14</value>
    </param>
```

```
    <param name="defaultFrgMinLen">
      <value>500</value>
    </param>
    <param name="moduleName">
      <value>P_AssembleUnitig</value>
    </param>
    <param name="ovlMinLen">
      <value>40</value>
    </param>
    <param name="libraryName">
      <value>pacbioReads</value>
    </param>
    <param name="specTmpl">
      <value>analysis/etc/celeraAssembler/unitig.spec</value>
    </param>
    <param name="description">
      <value>This module runs Celera Assembler v8.1 to the unitig step, then finishes
with our custom unitig consensus caller</value>
    </param>
    <param name="genomeSize">
      <value>12156677</value>
    </param>
    <param name="ovlErrorRate">
      <value>0.06</value>
    </param>
    <param name="xCoverage">
      <value>25</value>
    </param>
    <param name="maxSlotPerc">
      <value>1</value>
    </param>
  </module>
  <module name="P_ReferenceUploader">
    <param name="moduleName">
      <value>P_ReferenceUploader</value>
    </param>
    <param name="runUploaderUnitig">
      <value>True</value>
    </param>
    <param name="runUploaderHgap">
      <value>False</value>
    </param>
    <param name="sawriter">
      <value>sawriter -blt 8 -welter</value>
    </param>
    <param name="runUploader">
```

<value>False</value>
</param>
<param name="samIdx">
<value>samtools faidx</value>
</param>
<param name="name">
<value>reference</value>
</param>
</module>
<module name="P_Mapping">
<param name="maxHits">
<value>10</value>
</param>
<param name="maxDivergence">
<value>30</value>
</param>
<param name="placeRepeatsRandomly">
<value>True</value>
</param>
<param name="pulseMetrics">

<value>DeletionQV,IPD,InsertionQV,PulseWidth,QualityValue,MergeQV,SubstitutionQV
,DeletionTag</value>
</param>
<param name="pbalign_opts">
<value>--seed=1 --minAccuracy=0.75 --minLength=50 --algorithmOptions="-
useQuality"</value>
</param>
<param name="samBam">
<value>True</value>
</param>
<param name="moduleName">
<value>P_Mapping</value>
</param>
<param name="minAnchorSize">
<value>12</value>
</param>
<param name="description">
<value>BLASR maps reads to genomes by finding the highest scoring local alignment or set of local alignments between the read and the genome. The first set of alignments is found by querying an index of the reference genome, and then refining until only high scoring alignments are retained. Additional pulse metrics are loaded into the resulting cmp.h5 file to enable downstream use of the Quiver algorithm.</value>
</param>
<param name="loadPulsesOpts">
<value>bymetric</value>

```
    </param>
    <param name="gff2Bed">
        <value>True</value>
    </param>
  </module>
  <module name="P_MappingReports">
    <param name="moduleName">
        <value>P_MappingReports</value>
    </param>
  </module>
  <module name="P_AssemblyPolishing">
    <param name="moduleName">
        <value>P_AssemblyPolishing</value>
    </param>
    <param name="description">
        <value>Polish a pure-PacBio assembly for maximum accuracy using the Quiver
algorithm.</value>
    </param>
    <param name="enableMapQVFilter">
        <value>True</value>
    </param>
  </module>
</smrtpipeSettings>
```

PBJelly

```
#!/bin/bash -el

fasta="contigs.fa"
source $HOME/Programs/PBSuite_14.9.9/setup.sh

if [ ! -e reference ]; then
  mkdir reference
fi

sed s/\\./_/ $fasta > ./reference/reference.fasta

echo "setup"
Jelly.py setup Protocol.xml

echo "mapping"
Jelly.py mapping Protocol.xml

echo "support"
Jelly.py support Protocol.xml
```

```
echo "extraction"
Jelly.py extraction Protocol.xml

echo "assembly"
Jelly.py assembly Protocol.xml

echo "output"
Jelly.py output Protocol.xml
```

Protocol.xml

```
<jellyProtocol>
    <reference>./reference/reference.fasta</reference>
    <outputDir>pbjelly_out/</outputDir>
    <blasr>-minMatch 8 -minPctIdentity 70 -bestn 1 -nCandidates 20 -maxScore -500 -
nproc 4 -noSplitSubreads</blasr>
    <input baseDir="GLBRC-Data/Yeast/Y22-3/PacBio/">
        <job>Y22-3.pacbio.filtered_subreads.fastq</job>
    </input>
</jellyProtocol>
```

<u>Velvet</u>

```
#!/bin/bash -el


R1=GLBRCY22-3.R1.trimmed.fastq
R2=GLBRCY22-3.R2.trimmed.fastq


velveth=velveth
velvetg=velvetg

hash_list="45 47 49 51 53 55 57 59 61 63 65 67 69 71 73 75 77 79 81 83 85 87 89 91
93 95 97 99"

if [ ! -e results ]; then
  mkdir results
fi

for hash in $hash_list; do
  out_dir="results.$hash/"
  if [ ! -e velveth.$hash.run ]; then
    cmd="$velveth $out_dir $hash -fastq -shortPaired -separate $R1 $R2"
    echo $cmd
    $cmd >& velveth.$hash.log
    touch velveth.$hash.run
```

```
  fi

  contig="./results/contigs.h$hash.fa"
  if [ ! -e $contig ]; then
    cmd="$velvetg $out_dir -cov_cutoff auto -exp_cov auto"
    echo $cmd
    $cmd >& velvetg.h$hash.log
    cp $out_dir/contigs.fa $contig
  fi
done

echo "Done!"
```

Velvet-Bless

```
#Need untrimmed reads
R1=GLBRCY22-3_R1.fastq
R2=GLBRCY22-3_R2.fastq

mkdir k31
bless -read1 $R1 -read2 $R2 -prefix k31/Y22-3.bless -kmerlength 31

#Run velvet as above using
R1=./k31/Y22-3.bless.1.corrected.fastq
R2=./k31/Y22-3.bless.2.corrected.fastq
```

Velvet-Racer

```
racer=./RACER

#Can be either trimmed or raw reads
R1=GLBRCY22-3.R1.trimmed.fastq
R2=GLBRCY22-3.R2.trimmed.fastq

genome_size=12156677

$racer $R1 RACER.R1.fastq $genome_size
$racer $R2 RACER.R2.fastq $genome_size
#Run velvet as above using
R1=RACER.R1.fastq
R2=RACER.R2.fastq
```
