# Supplementary material for: Genome Sequence and Analysis of a Stress-Tolerant, Wild-Derived Strain of Saccharomyces cerevisiae Used in Biofuels Research
Source: G3 (Bethesda). 2016 Apr 16;6(6):1757–66. doi: 10.1534/g3.116.029389 (PMC4889671; doi:10.1534/g3.116.029389)
Supplement: Supplemental Material [file supp_g3.116.029389_FileS3.pdf]

FILE S3: SUPPLEMENTARY RESULTS

Mitochondrial genome sequence and structure: The size of the Y22-3 mitochondrial genome is 82897 bp, which is 2.8 kbp smaller than the S288c mitochondrial genome. Y22-3 has 24 mitochondrial tRNA annotations and a similar gene order to S288c, but it differs in the number of introns in *COX1,* the positions of GC clusters*,* and the presence of several free-standing homing endonucleases and maturases (**Figure S5**). Specifically, the first four exons of *COX1* are combined into a unique exon in Y22-3 such that the *COX1* gene contains four introns, instead of the seven found in S288c. Although not previously noted, the unusual exon configuration of Y22-3 *COX1* is also present in two clinical *S. cerevisiae* strains, YJM1549 and YJM1332 (Wolters *et al.* 2015). Intron 2 of *COX1* encodes a maturase, ai4β, which is similar to a maturase found in the clinical *S. cerevisiae* isolate YJM789 (Wei *et al.* 2007) and 19 Mosaic-A, four wine/European, and 15 Mosaic-B *S. cerevisiae* strains (Wolters *et al.* 2015). Although this intron and its maturase exist in only 38 out of 103 *S. cerevisiae* strains, ai4β also exists in *Saccharomyces pastorianus* W34/70 (Nakao *et al.* 2009).

Y22-3 contains two putative homing endonucleases not found in S288c, homologs of which have been described previously in other *S. cerevisiae* strains, such as CBS 2354 (= NCYC 74) and D273-10B, as *RF2* and *RF3*, respectively (Michel 1984; Séraphin *et al.* 1985, 1987). Although not annotated in the recent analysis of 103 *S. cerevisiae* mitochondrial genomes (Wolters *et al.* 2015), we found *RF2* in NCYC3594 and YJM1439, and we found *RF3* in YJM789, YJM1078, and YJM1273, suggesting that their presence in Y22-3 is rare but not unique. Along with *ORF1*, which is present in

S288c, these homing endonucleases are members of the LAGLIDADG family. Y22-3 *ORF1* has no premature stop codons or GC clusters shifting *ORF1* frame, so it may be active, as has been suggested for other *Saccharomyces* strains (Peris *et al.* 2015).

A gene sequence comparison, using the coding sequences of *COX1, COX2, COX3, ATP6, ATP8, ATP9, COB*, and *VAR1* showed that the mitochondrial genomes of Y22-3 and S288c were surprisingly divergent with 1.23% nucleotide differences between them, which is only slightly less than the divergences between Y22-3 and the reference genome of the sister species, *Saccharomyces paradoxus* CBS 432 (2.22%), or *S. pastorianus* W34/70 (6.35%). High diversity (1.07%) among 103 *S. cerevisiae* mitochondrial coding sequences has recently been noted (Wolters *et al.* 2015). For Y22-3 and for *S. cerevisiae* more broadly, the high nucleotide diversity is mainly driven by *COX2* and *VAR1*. High nucleotide diversity at *COX2* has been suggested to be the result of reticulate evolution (e.g. recombination, introgression, horizontal gene transfer) mediated by *ORF1*, GC clusters, and AT tandem repeat regions (Peris *et al.* 2015).

LITERATURE CITED

Michel, F., 1984 A maturase-like coding sequence downstream of the *OXI2* gene of yeast mitochondrial DNA is interrupted by two GC clusters and a putative end-of-messenger signal. Curr Genet 8: 307–317.

Nakao, Y., T. Kanamori, T. Itoh, Y. Kodama, S. Rainieri *et al.*, 2009 Genome Sequence of the Lager Brewing Yeast, an Interspecies Hybrid. DNA Res 16: 115–129.

Peris, D., A. Arias, S. Orlic, C. Belloch, L. Perez-Traves *et al.*, 2015 Mitochondrial introgression suggests extensive ancestral hybridization events among *Saccharomyces* species. bioRxiv 10.1101: 028324.

Séraphin, B., M. Simon, and G. Faye, 1985 A mitochondrial reading frame which may code for a maturase-like protein in *Saccharomyces cerevisiae*. Nucleic Acids Res 13: 3005–3014.

Séraphin, B., M. Simon, and G. Faye, 1987 The mitochondrial reading frame *RF3* is a functional gene in *Saccharomyces uvarum*. J Biol Chem 262: 10146–10153.

Wei, W., J. H. McCusker, R. W. Hyman, T. Jones, Y. Ning *et al.*, 2007 Genome sequencing and comparative analysis of *Saccharomyces cerevisiae* strain YJM789. Proc Natl Acad Sci 104: 12825–12830.

Wolters, J. F., K. Chiu, and H. L. Fiumera, 2015 Population structure of mitochondrial genomes in *Saccharomyces cerevisiae*. BMC Genomics 16: 451.
