# Supplementary figures and images for: Genome Sequence and Analysis of a Stress-Tolerant, Wild-Derived Strain of Saccharomyces cerevisiae Used in Biofuels Research
Source: G3 (Bethesda). 2016 Apr 16;6(6):1757–66. doi: 10.1534/g3.116.029389 (PMC4889671; doi:10.1534/g3.116.029389)

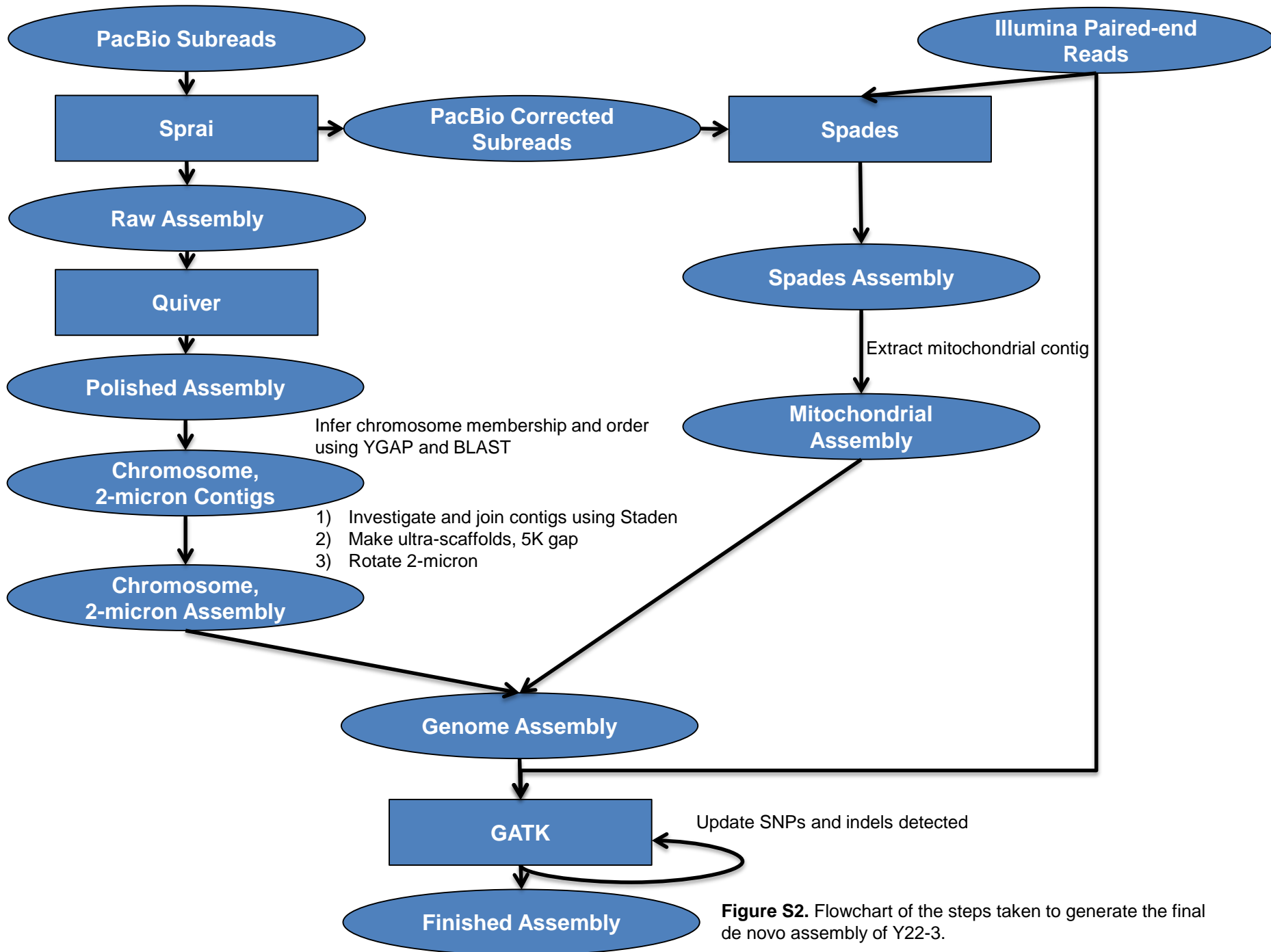

Supplement: Supplemental Material [file supp_g3.116.029389_FigureS2.pdf]

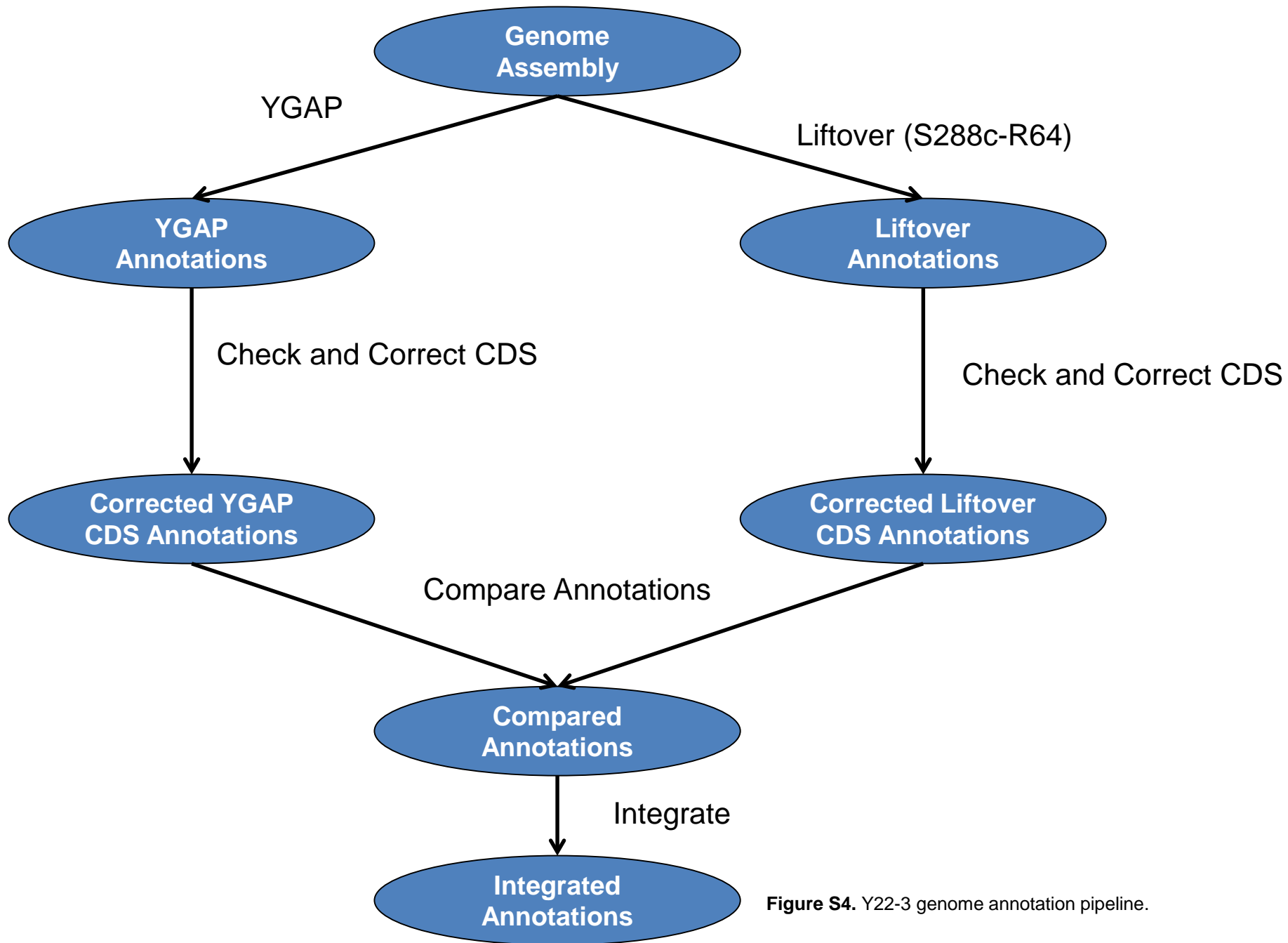

**Figure S4.** Y22-3 genome annotation pipeline.

Supplement: Supplemental Material [file supp_g3.116.029389_FigureS4.pdf]
