# Supplementary material for: Genome Sequence and Analysis of a Stress-Tolerant, Wild-Derived Strain of Saccharomyces cerevisiae Used in Biofuels Research
Source: G3 (Bethesda). 2016 Apr 16;6(6):1757–66. doi: 10.1534/g3.116.029389 (PMC4889671; doi:10.1534/g3.116.029389)
Supplement: Supplemental Material [file supp_g3.116.029389_FigureS3.pdf]

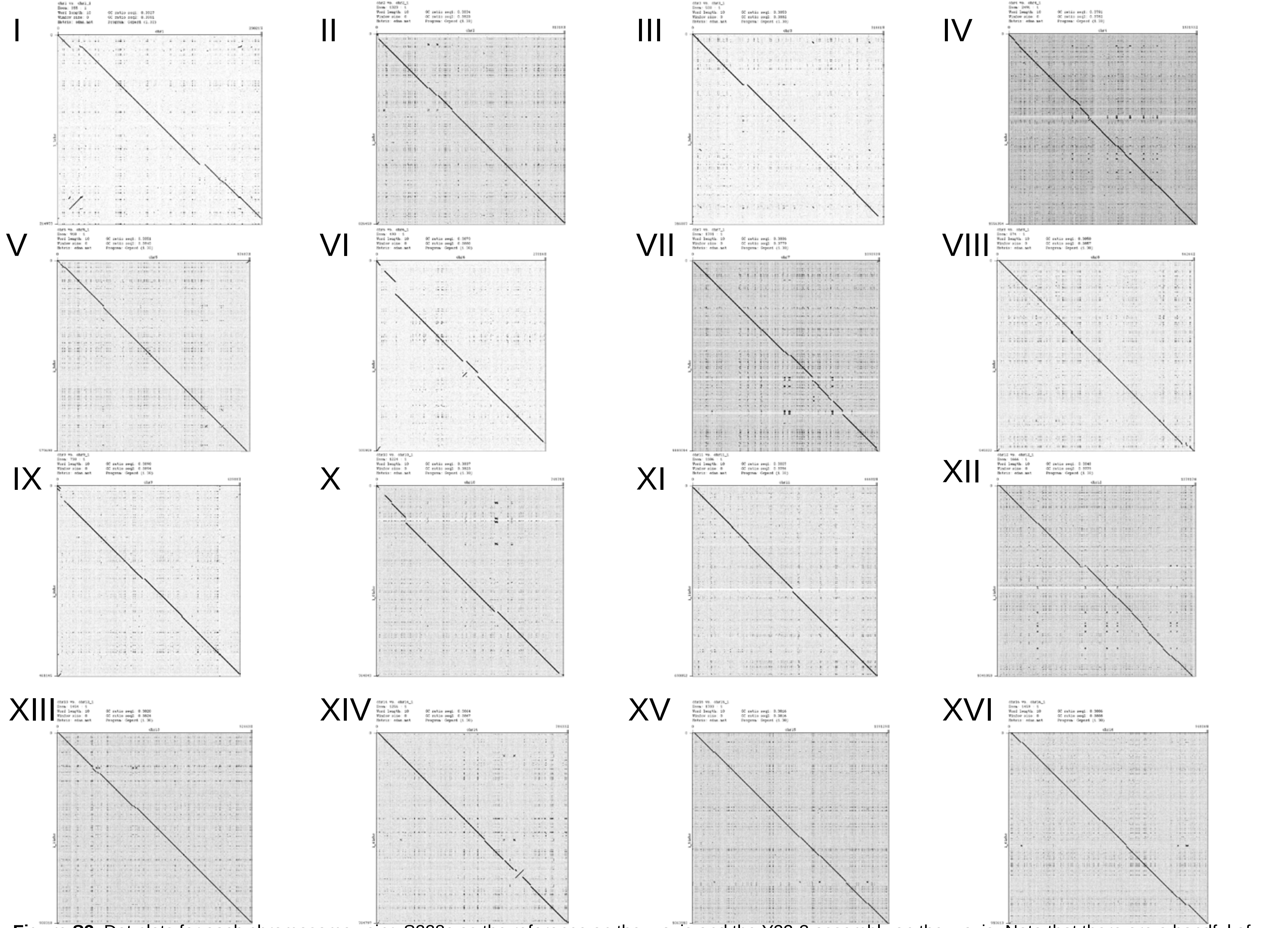


**Figure S3.** Dot plots for each chromosome using S228c as the reference on the x-axis and the Y22-3 assembly on the y-axis. Note that there are a handful of small inversions but no detectable translocations.
