# Supplementary material for: Genome Sequence and Analysis of a Stress-Tolerant, Wild-Derived Strain of Saccharomyces cerevisiae Used in Biofuels Research
Source: G3 (Bethesda). 2016 Apr 16;6(6):1757–66. doi: 10.1534/g3.116.029389 (PMC4889671; doi:10.1534/g3.116.029389)
Supplement: Supplemental Material [file supp_g3.116.029389_FigureS5.pdf]

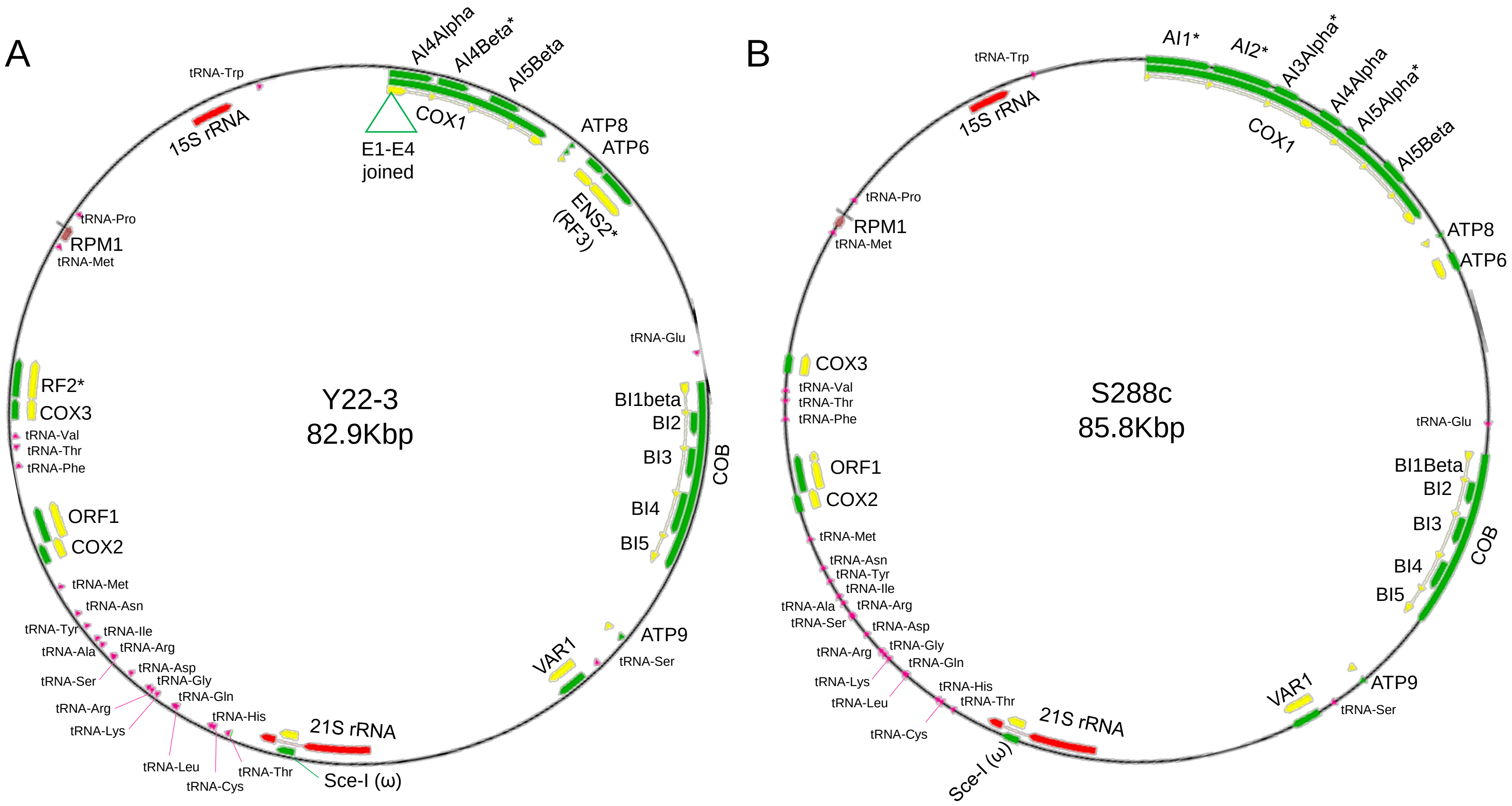

Figure S5. Schematic representation of A) Y22-3 and B) S288c mitochondrial genome annotations. Mitochondrial protein-coding genes, including their introns; coding sequences (CDS); rRNAs; tRNAs; and non-coding RNAs are represented in green, yellow, red, pink, and brown, respectively. Genes with asterisks are additional coding sequences in each mitochondria. The triangle in the *COX1* gene represents the fusion of exons 1 to 4 into an exon in Y22-3 that S288c lacks.
