# Supplementary material for: Genome Sequence and Analysis of a Stress-Tolerant, Wild-Derived Strain of Saccharomyces cerevisiae Used in Biofuels Research
Source: G3 (Bethesda). 2016 Apr 16;6(6):1757–66. doi: 10.1534/g3.116.029389 (PMC4889671; doi:10.1534/g3.116.029389)
Supplement: Supplemental Material [file supp_g3.116.029389_FigureS6.pdf]

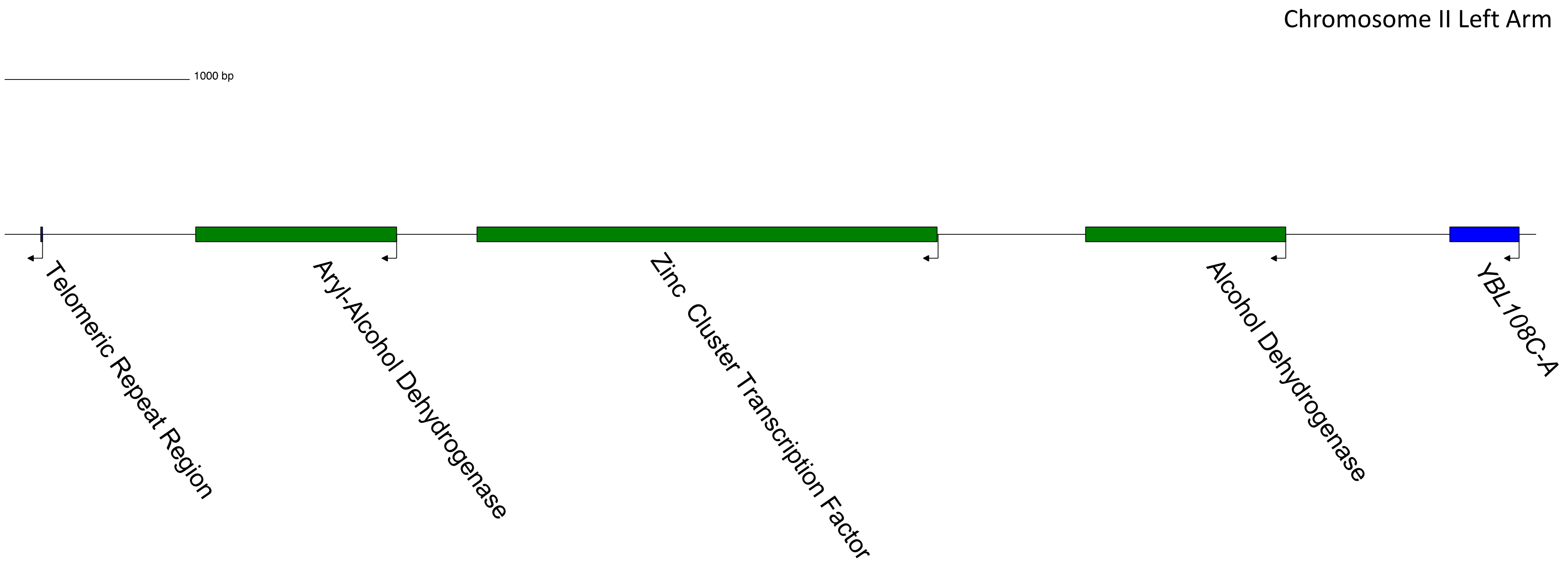

Figure S6. GenePalette depiction of novel genes and non-syntenic homologs found in the subtelomeric region of the left arm of chromosome II. Ψ, pseudogene. Features syntenic with S288c are in blue, and novel genes and non-syntenic homologs with valid coding regions are in green, and pseudogenes are in red. The scale bar represents 1000 bp.
