# Supplementary material for: Genome Sequence and Analysis of a Stress-Tolerant, Wild-Derived Strain of Saccharomyces cerevisiae Used in Biofuels Research
Source: G3 (Bethesda). 2016 Apr 16;6(6):1757–66. doi: 10.1534/g3.116.029389 (PMC4889671; doi:10.1534/g3.116.029389)
Supplement: Supplemental Material [file supp_g3.116.029389_FigureS1.pdf]

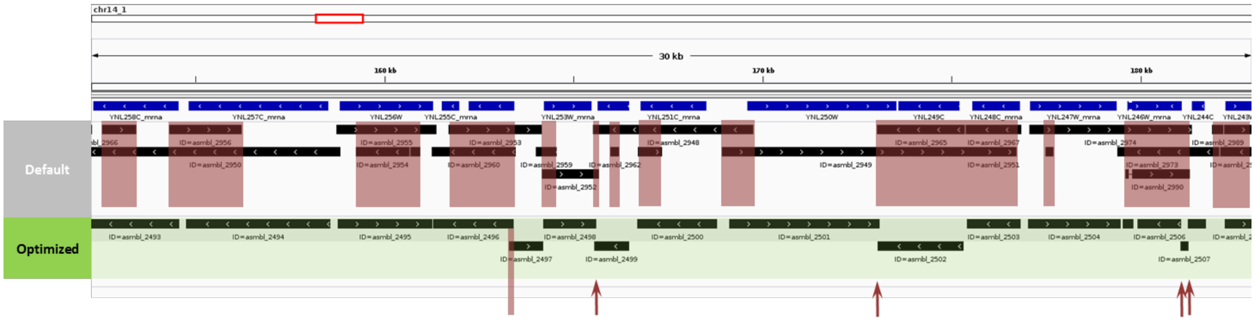

Figure S1. A sample region from chromosome XIV demonstrating the predicted genes (blue track), the alignment of the predicted transcripts generated with default-parameter Trinity assembly (grey zone), and the alignment of the predicted transcripts generated with the optimized Trinity assembly (green zone). Blocks, zones of both-strand coverage by predicted transcripts; arrows, zone of both-strand coverage in the optimized assembly that are too narrow (several nucleotide pairs) for block highlighting. For both Trinity assemblies, the genome sequence was not used to assist in the transcript assembly, so the de novo transcriptome provides an independent validation of the predicted protein-coding genes.
